# Single-Index Mixed-Effects Model for Asymmetric Bivariate Clustered Data

**DOI:** 10.1007/s41096-024-00181-0

**Published:** 2024-03-16

**Authors:** Weihua Zhao, Dipankar Bandyopadhyay, Heng Lian

**Affiliations:** 1 School of Sciences, Nantong University, Nantong, China; 2 Department of Mathematics, City University of Hong Kong, Kowloon Tong, Hong Kong, China; 3 Department of Biostatistics, School of Population Health, Virginia Commonwealth University, Richmond, VA, USA

**Keywords:** Asymmetric Laplace distribution, Clustered data, EM algorithm, Random-effects, Single-index model

## Abstract

Studies/trials assessing status and progression of periodontal disease (PD) usually focus on quantifying the relationship between the clustered (tooth within subjects) bivariate endpoints, such as probed pocket depth (PPD), and clinical attachment level (CAL) with the covariates. Although assumptions of multivariate normality can be invoked for the random terms (random effects and errors) under a linear mixed model (LMM) framework, violations of those assumptions may lead to imprecise inference. Furthermore, the response-covariate relationship may not be linear, as assumed under a LMM fit, and the regression estimates obtained therein do not provide an overall summary of the risk of PD, as obtained from the covariates. Motivated by a PD study on Gullah-speaking African-American Type-2 diabetics, we cast the asymmetric clustered bivariate (PPD and CAL) responses into a non-linear mixed model framework, where both random terms follow the multivariate asymmetric Laplace distribution (ALD). In order to provide a one-number risk summary, the possible non-linearity in the relationship is modeled via a single-index model, powered by polynomial spline approximations for index functions, and the normal mixture expression for ALD. To proceed with a maximum-likelihood inferential setup, we devise an elegant EM-type algorithm. Moreover, the large sample theoretical properties are established under some mild conditions. Simulation studies using synthetic data generated under a variety of scenarios were used to study the finite-sample properties of our estimators, and demonstrate that our proposed model and estimation algorithm can efficiently handle asymmetric, heavy-tailed data, with outliers. Finally, we illustrate our proposed methodology via application to the motivating PD study.

## 1 Introduction

Epidemiological studies in a clustered, or longitudinal data setting often generate multivariate (repeated) outcomes that are analyzed under the ubiquitous multivariate normal (MVN) assumptions of the random terms (random effects, and within-subject random errors) via standard software, such as SAS, or R. However, violations of those assumptions can lead to imprecise parameter estimates (Bandyopadhyay et al. 2010). These non-Gaussian features are usually manifested through skewness of the response vector, and/or thick-tails. Although achieving close-to-normality via suitable data transformations of the responses (such as log, or Box-Cox) for standard linear mixed model (LMM) analysis are possible, they maybe avoided due to their non-universality, and difficulty in covariate interpretation on the original scale (Jara et al. 2008). To address this, various flexible (parametric) alternatives to the MVN density exists, such as the multivariate skewnormal density (Azzalini and Capitanio 1999; Gupta et al. 2004; Azzalini 2010), the heavy-tailed multivariate skew *t*-density (Azzalini and Capitanio 2003), and others, that can accommodate departures from normality without resorting to adhoc data transformations.

In practice, this setup can be further complicated in presence of multiple outcomes recorded at each cluster units/components. The motivating data example in this paper comes from a clinical study of periodontal disease (PD) conducted on Gullah-speaking African-American Type-2 diabetics (henceforth, GAAD). Here, the multiple outcomes of interest are the *tooth-level* (mean) probed pocket depth (PPD) and clinical attachment level (CAL), which are recorded (in mm, via a periodontal probe) simultaneously for each tooth nested/clustered within a subject. While PPD quantifies the current PD status, CAL measures the (past) disease history and progression (Page and Eke 2007). An oral clinician may be interested in studying the *joint* evolution of these outcomes over some features of covariates, and the complexity is induced from two different sources of correlation—(a) Between repeated observations of any given outcome (PPD, or CAL) measured at a cluster unit (tooth), and (b) Between multiple outcomes (PPD and CAL) measured at the same tooth. The existing literature (both classical and Bayesian) in this context of multiple repeated outcomes modeling is also very rich (Luo and Wang 2014; Verbeke et al. 2014; Lin and Wang 2013; Michaelis et al. 2018; Bandyopadhyay et al. 2010). However, a vast majority of these models are developed under the *restrictive* assumption of linearity of the covariate effects over the multivariate responses.

**Fig. 1:**
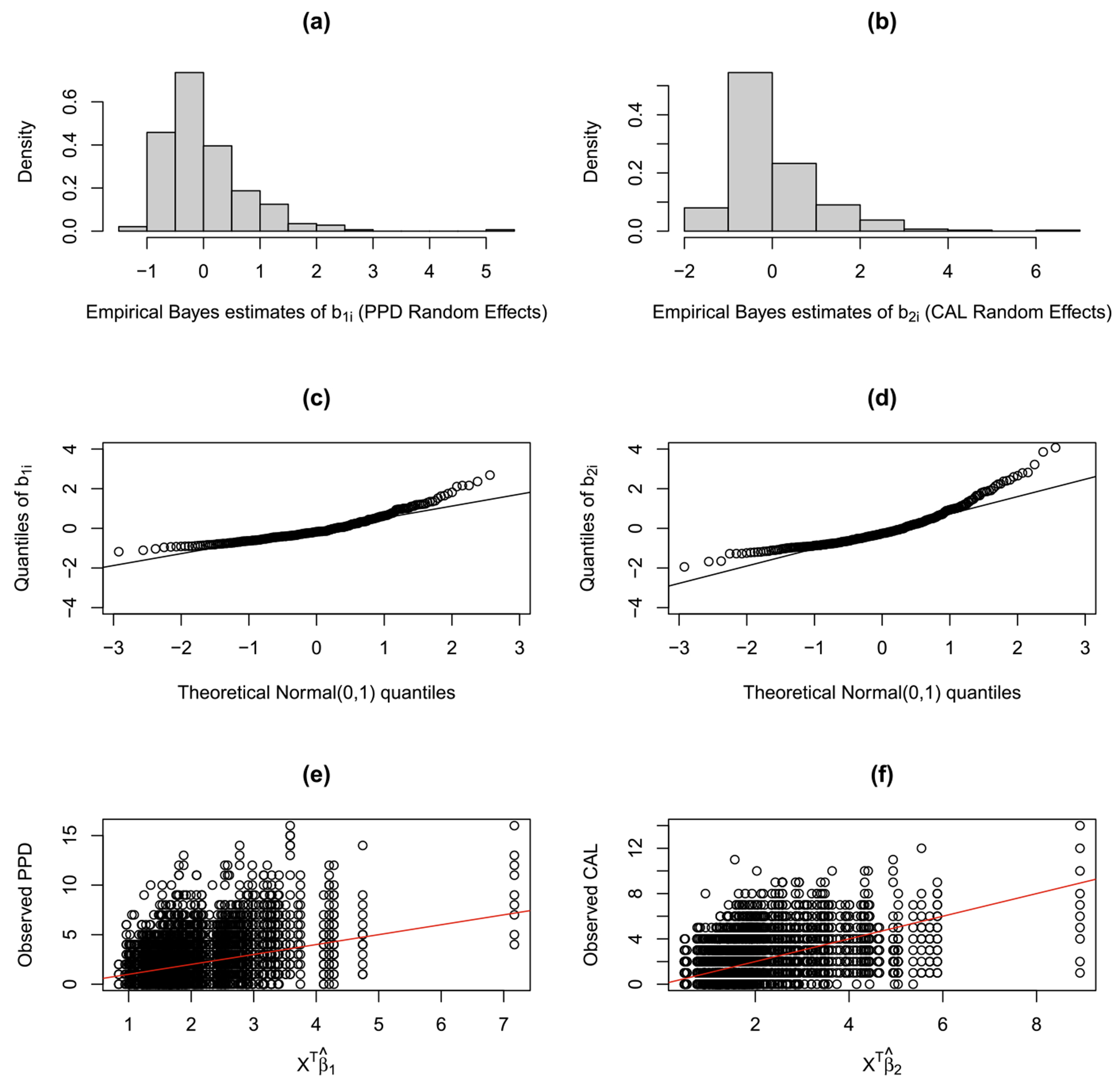
GAAD Data: Plots of the empirical Bayes’ estimates of random effects (panels a and b), corresponding Q-Q plots (panels c and d), and observed versus estimated (non-linear) curve (panels e and f), obtained from fitting a linear mixed model separately to the PPD and CAL responses

To motivate further, consider Fig. 1, which presents plots of the empirical Bayes’ estimates of random effects (panels a and b), corresponding Q-Q plots (panels c and d), and observed versus estimated (non-linear) curve (panels e and f), obtained from fitting a LMM separately to the PPD and CAL responses in the GAAD data, using the lme function in R. The plots clearly reveal evidence of asymmetryq (departures from the Gaussian assumptions), which cannot be explained by a standard LMM fit. In addition, the predictor space restricted to be linear combinations of covariates may not provide an elegant picture of their cross-sectional association with the (bivariate) response. Formulating an *index* for PD (that handles possible nonlinearity, confounding, and interaction effects between the PD outcomes and the covariates) via a single-index model, or SIM (Hardle et al. 1993) can be a clinically elegant alternative. SIMs are a popular class of semiparametric regression models that relaxes the assumption of linearity, and bypass the ‘curse of dimensionality’ by reducing the multi-dimensional predictor space X into an univariate (scalar) index $U=X^{T}\;\beta$. A link function *g*(.) now connects the covariate space to the response Y, offering a pragmatic compromise between a fully nonparametric (and often non-interpretable) multiple regression, and a restrictive (parametric) linear regression. Here, the magnitude of the index coefficient $\beta_{j}$ determine the relative importance of the j-th predictor on the index, and g(U) denotes the location of interest in the response curve at the index U. In biomedical research, the recent work by Wu and Tu (2016) develops an adiposity index via a (multivariate) SIM to efficiently predict multiple longitudinal outcomes (systolic and diastolic blood pressure) in children. However, their proposal considers the usual MVN assumptions for the random terms (errors and effects), and may not well accommodate heavy tailed and other non-Gaussian features. Furthermore, they did not provide rigorous theoretical justification.

Considering Wu and Tu (2016) as our starting point, we seek to develop an index that can efficiently predict the clustered bivariate (PPD and CAL) PD outcomes. Such a *clinical* index that links both outcomes is vastly absent in the oral health literature. Our bivariate single-index mixed (BV-SIM) model tackles non-Gaussian features in the responses via the multivariate asymmetric Laplace density (ALD; Kotz et al. 2001) assumptions in the random terms. The multivariate ALD can accommodate asymmetric, peaked, and heavy-tailed data using fewer number of parameters than the popular multivariate skew-*t* density (Gupta 2003). The multivariate symmetric Laplace density (Naik and Plungpongpun 2006), a special case of the ALD, has been applied in other fields, such as speech clustering, classification problems, and image/signal analysis. Under this framework, we consider a polynomial spline approximation to the nonparametric index function, and propose an efficient EM-type algorithm for estimation and inference. The spline approximation, and the mixture normal representation of the multivariate ALD presents a computationally efficient, and intuitively appealing estimation setup, quantifying correlations from both sources.

The rest of the paper is organized as follows. In Sect. 2, we propose the BV-SIM model under the assumptions of a multivariate asymmetric Laplace density. Using the polynomial splines approximation for the nonparametric (index) functions, we derive the maximum likelihood (ML) estimate, and establish the large sample properties of the proposed estimators in Sect. 3, with the detailed technical proofs relegated to the Appendix, where we use the projection method to prove the asymptotic normality of parametric part. In Sect. 4, we develop an efficient MLE procedure based on the EM-algorithm. Simulation studies comparing finite sample performance of our approach to other alternatives appear in Sect. 5, while Sect. 6 illustrates the method via application to the PD dataset. Finally, some concluding remarks are presented in Sect. 7.

## 2 Statistical Model

We begin with a sketch of the multivariate shifted Laplace density (Kotz et al. 2001), and then develop our SIM mixed effects framework for bivariate clustered data. The multivariate ALD has the density

(2.1) $$p(y;\Sigma ,\gamma )=\frac{2\;\text{exp}\left\{y^{\text{T}}\Sigma^{-1}\gamma \right\}}{{\left(2\pi \right)}^{d/2}|\Sigma {|}^{1/2}}\times {\left(\frac{y^{\text{T}}\Sigma^{-1}y}{2+\gamma^{\text{T}}\Sigma^{-1}\gamma }\right)}^{\nu /2}K_{\nu }(u),$$

where $K_{\nu }$ is the modified Bessel function of the third kind with index ν, $\nu =(2-d)/2,u=\sqrt{\left(2+\gamma^{T}\Sigma^{-1}\gamma \right)\left(y^{T}\Sigma^{-1}y\right)},\gamma \in {\mathbb{R}}^{d}$ is a skewness parameter and Σ is a positive definite (p.d.) scatter matrix with dimension d×d. We denote (2.1) as ${ALD}_{d}(\Sigma ,\gamma )$. Note, the ALD forces each component density to be joined at the same origin. An extension, the multivariate shifted asymmetric Laplace distribution (SALD; Kotz et al. 2001), has the form

(2.2) $$p(y;\mu ,\Sigma ,\gamma )=\frac{2\;\text{exp}\left\{{\left(y-\mu \right)}^{\text{T}}\Sigma^{-1}\gamma \right\}}{{\left(2\pi \right)}^{d/2}|\Sigma {|}^{1/2}}\times {\left(\frac{\delta (y,\mu ,\Sigma )}{2+\gamma^{\text{T}}\Sigma^{-1}\gamma }\right)}^{\nu /2}K_{\nu }(u),$$

where $u=\sqrt{\left(2+\gamma^{T}\Sigma^{-1}\gamma \right)\delta (y,\mu ,\Sigma )},\delta (y,\mu ,\Sigma )=(y-\mu {)}^{T}\Sigma^{-1}(y-\mu )$, and ν,γ,Σ are defined in (2.1). Here, we use the notation $Y∼{SAL}_{d}(\mu ,\Sigma ,\gamma )$ to denote the random variable y following a d-dimensional SALD. After some calculations, the mean and variance of SALD are given by

$$E(Y)=\mu +\gamma \;\text{and}\;Var(Y)=\Sigma +\gamma \gamma^{T}.$$

It is clear that the mean depends on the shifted location parameter μ and skewness parameter γ, while its variance depends on scatter matrix Σ and skewness parameter γ. Also, $\Sigma +\gamma \gamma^{T}$ must be p.d. if Σ is p.d. The parameter γ plays an important role in multivariate asymmetric data analysis, besides the location μ and scatter matrix Σ. Note, the multivariate density in (2.2) reduces to (2.1) when μ=0, and it further reduces to the multivariate symmetry Laplace distribution (Eltoft et al. 2006) when γ=0. Moreover, (2.2) reduces to the univariate ALD when dimension d=1,γ=(1−2τ)/τ(1−τ) and $\Sigma_{1\times 1}=2/\tau (1-\tau )$, and is popularly used in the likelihood framework for quantile regression with density $p(y)=\tau (1-\tau )exp\left\{-\rho_{\tau }(y-\mu )\right\}$, where $\rho_{\tau }(u)=u(\tau -I(u<0))$. The SALD in (2.2) has the following stochastic representation

(2.3) $$Y=\mu +V\gamma +\sqrt{V}Z,$$

where V is a random variable from an exponential distribution with mean 1 and $Z∼N_{d}(0,\Sigma )$ is generated independent of V. Using Bayes’s theorem, the density of V given Y=y is generalized inverse Gaussian, with the density

(2.4) $$p_{V}(\nu |Y=y)=\frac{\nu^{\nu -1}}{2K_{\nu }(u)}{\left(\frac{\delta (y,\mu ,\Sigma )}{2+\gamma^{\text{T}}\Sigma^{-1}\gamma }\right)}^{-\nu /2}\text{exp}\left\{-\frac{1}{2\nu }\delta (y,\mu ,\Sigma )-\frac{\nu }{2}\left(2+\gamma^{\text{T}}\Sigma^{-1}\gamma \right)\right\},$$

where ν,γ,μ,Σ,δ(y,μ,Σ) and u are as defined in (2.2). The SALD allows for peakedness, heavy tails, and skewness, and hence provides more flexibility in modeling multivariate data with non-Gaussian features. More properties, extensions and applications of SALD appear in Kozubowski and Podgórski (2001); Franczak et al. (2014); Bouveyron and Brunet-Saumard (2014).

### 2.1 Single-Index Mixed-Effects Model

Let $y_{ij}={\left(y_{ij}^{\left(1\right)},y_{ij}^{\left(2\right)}\right)}^{T}$ be the observed values of two response variables (here, mean PPD and CAL) for the ith subject at the jth location (here, tooth), where i=1,…,n and $j=1,\ldots ,m_{i}$. We assume

(2.5) $$\left\{y_{ij}={\tilde{\mu }}_{ij}+\epsilon_{ij},\;{\tilde{\mu }}_{ij}={\left({\tilde{\mu }}_{ij}^{\left(1\right)},{\tilde{\mu }}_{ij}^{\left(2\right)}\right)}^{T},\;{\tilde{\mu }}_{ij}^{\left(1\right)}=g_{1}\left({\left(x_{ij}^{\left(1\right)}\right)}^{T}\beta_{1}\right)+{\left(z_{ij}^{\left(1\right)}\right)}^{T}b_{i1},\;{\tilde{\mu }}_{ij}^{\left(2\right)}=g_{2}\left({\left(x_{ij}^{\left(2\right)}\right)}^{T}\beta_{2}\right)+{\left(z_{ij}^{\left(2\right)}\right)}^{T}b_{i2},\;\epsilon_{ij}∼{SAL}_{2}(0,\Sigma ,\gamma ),\mathrm{i.i.d.}\;\forall i,j\right.,$$

where $g_{1}$ and $g_{2}$ are two unknown nonparametric functions, $x_{ij}^{\left(1\right)}={\left(x_{ij1}^{\left(1\right)},\ldots ,x_{ijp_{1}}^{\left(1\right)}\right)}^{T}$, $x_{ij}^{\left(2\right)}={\left(x_{ij1}^{\left(2\right)},\ldots ,x_{ijp_{2}}^{\left(2\right)}\right)}^{T},$ and $z_{ij}^{\left(1\right)}={\left(1,z_{ij1}^{\left(1\right)},\ldots ,z_{ijq_{1}}^{\left(1\right)}\right)}^{T},\;z_{ij}^{\left(2\right)}={\left(1,z_{ij1}^{\left(2\right)},\ldots ,z_{ijq_{2}}^{\left(2\right)}\right)}^{T}$, $\beta_{k}\in {\mathbb{R}}^{p_{k}}$ and $b_{ik}\in {\mathbb{R}}^{q_{k}+1}$ are the (fixed) index coefficients and random effect for the k-th response (k=1or2), γ is a 2×1 vector of skewness parameters, and Σ is the scatter matrix with dimension 2×2 for the random error ϵ. To accommodate a robust specification, we also assume the random effects $b_{i}={\left({b_{i1}}^{T},{b_{i2}}^{T}\right)}^{T}∼{SAL}_{\left(q_{1}+q_{2}+2\right)}(0,\Omega ,0)$, where Ω is an unstructured covariance matrix with dimension $\left(q_{1}+q_{2}+2\right)\times \left(q_{1}+q_{2}+2\right)$. Note, Ω carries information pertaining to both the clustering correlation within a response found on the two blocks of diagonal sub-matrices, with dimensions $\left(q_{1}+1\right)\times \left(q_{1}+1\right)$ and $\left(q_{2}+1\right)\times \left(q_{2}+1\right)$, and the cross-correlations between responses, found on the off-diagonal sub-matrices. In addition, we further assume the joint density of ${\left({\epsilon_{ij}}^{T},{b_{i}}^{T}\right)}^{T}$ is ${SAL}_{\left(q_{1}+q_{2}+4\right)}\left(0_{\left(q_{1}+q_{2}+4\right)}\right.$, $\left.\mathrm{blockdiag}(\Sigma ,\Omega ),{\left(\gamma^{T},0_{q_{1}+q_{2}+4}^{T}\right)}^{T}\right)$. We call model (2.5) as the single-index mixed-effects (SIME) model for bivariate clustered data.

For identifiability, we assume both $∥\beta_{1}∥=1$ and $∥\beta_{2}∥=1$, and their first components are positive, respectively. In this paper, the popular “delete one component” method is used to avoid the equality constraints (Yu and Ruppert 2002; Cui et al. 2011). Specifically, we write $\beta_{1}={\left({\left(1-{∥\beta_{1}^{\left(-1\right)}∥}^{2}\right)}^{1/2},\beta_{12},\ldots ,\beta_{1p_{1}}\right)}^{T}$ where, $\beta_{1}^{\left(-1\right)}={\left(\beta_{12},\ldots ,\beta_{1p_{1}}\right)}^{T}$. Under this parametrization, $\beta_{1}$ is a smooth deterministic function of $\beta_{1}^{\left(-1\right)}$, with its Jacobian matrix given by

$$J_{1}=\frac{\partial \beta_{1}}{\partial \beta_{1}^{\left(-1\right)}}=\left(\begin{array}{c} -\frac{\beta_{1}^{\left(-1\right)}}{{\left(1-{∥\beta_{1}^{\left(-1\right)}∥}^{2}\right)}^{1/2}}\\ I_{p_{1}-1} \end{array},\right)$$

where $I_{p_{1}-1}$ is the identity matrix with $p_{1}-1$ rows/columns. The true parameter $\beta_{1}^{\left(-1\right)}$ satisfies the constraint $\beta_{1}^{\left(-1\right)}<1$, which implies that it is a interior point in a unit ball in ${\mathbb{R}}^{p_{1}-1}$. Therefore, $\beta_{1}$ is infinitely differentiable in a neighborhood of $\beta_{1}^{\left(-1\right)}$. Similarly, we define $\beta_{2}^{\left(-1\right)}$ and $J_{2}$, and let $\beta^{\left(-1\right)}={\left({\left(\beta_{1}^{\left(-1\right)}\right)}^{T},{\left(\beta_{2}^{\left(-1\right)}\right)}^{T}\right)}^{T}$, $J=\mathrm{blockdiag}\left(J_{1},J_{2}\right)$. Applying the stochastic representation in (2.3), model (2.5) admits the following hierarchical structure:

(2.6) $$\left\{\begin{array}{l} y_{i}|b_{i},V_{i}∼N_{2m_{i}}\left({\tilde{\mu }}_{i}+V_{i}\left(1_{m_{i}}⊗\gamma \right),V_{i}\Lambda_{i}\right),\\ b_{i}|V_{i}∼N_{2(q+1)}\left(0,V_{i}\Omega \right),\;V_{i}∼E(1), \end{array}\right.$$

where $y_{i}={\left({y_{i1}}^{T},\ldots ,{y_{im_{i}}}^{T}\right)}^{T},{\tilde{\mu }}_{i}={\left({{\tilde{\mu }}_{i1}}^{T},\ldots ,{{\tilde{\mu }}_{im_{i}}}^{T}\right)}^{T}$, E denotes the exponential distribution, $\Lambda_{i}=I_{m_{i}}⊗\Sigma$, where ⊗ denotes the kronecker product, and $1_{m_{i}}$ is a $m_{i}$ column vector with element 1 . From (2.5) and (2.6), it is clear that conditional on $V_{i},\epsilon_{ij}$ and $b_{i}$ are independent. Integrating out $b_{i}$ in (2.6), we have the following hierarchical model

(2.7) $$y_{i}|V_{i}∼N_{2m_{i}}\left(\mu_{i}+V_{i}\left(1_{m_{i}}⊗\gamma \right),V_{i}G_{i}\right),\;V_{i}∼E(1),$$

where $\mu_{i}={\left({\left(\mu_{i1}\right)}^{T},\ldots ,{\left(\mu_{im_{i}}\right)}^{T}\right)}^{T}$ with $\mu_{ij}={\left(g_{1}\left({\left(x_{ij}^{\left(1\right)}\right)}^{T}\beta_{1}\right),g_{2}\left({\left(x_{ij}^{\left(2\right)}\right)}^{T}\beta_{2}\right)\right)}^{T}$, $Z_{i}=\left(Z_{i1},\ldots ,Z_{im_{i}}\right),Z_{ij}=\mathrm{blockdiag}\left(z_{ij}^{\left(1\right)},z_{ij}^{\left(2\right)}\right),G_{i}={Z_{i}}^{T}\Omega Z_{i}+\Lambda_{i}$. Moreover, it follows from (2.7) that the $y_{i}$ are independent and marginally distributed as

(2.8) $$y_{i}∼{SALD}_{2m_{i}}\left(\mu_{i},G_{i},\gamma_{i}^{*}\right),\;i=1,\ldots ,n,$$

where $\gamma_{i}^{*}=1_{m_{i}}⊗\gamma$. From (2.7) and by the properties of the generalized inverse Gaussian distribution in (2.4), we have

(2.9) $$𝔼\left(V_{i}|y_{i}\right)=\sqrt{\frac{b_{i}}{a_{i}}}R_{\nu }\left(\sqrt{a_{i}b_{i}}\right)\;\text{and}\;𝔼\left(V_{i}^{-1}|y_{i}\right)=\sqrt{\frac{a_{i}}{b_{i}}}R_{\nu }\left(\sqrt{a_{i}b_{i}}\right)-\frac{2\nu }{b_{i}},$$

where $a_{i}=2+{\left(\gamma_{i}^{*}\right)}^{T}G_{i}^{-1}\gamma_{i}^{*},\;b_{i}={\left(y_{i}-\mu_{i}\right)}^{T}G_{i}^{-1}\left(y_{i}-\mu_{i}\right),\;R_{\nu }(u)=K_{\nu +1}(u)/K_{\nu }(u)$ and $\nu =1-m_{i}$.

### 2.2 Modeling the Index Functions

Since the two functions $g_{1}$ and $g_{2}$ in (2.5) are unknown, we use polynomial splines to approximate them in the subsequent ML estimation. Polynomial splines are simple, yet practical tools with computational tractability and statistical efficiency, and has been proven to be an extremely powerful method for smoothing.

For simplicity, we assume that the covariates $x_{ij}^{\left(1\right)}$ and $x_{ij}^{\left(2\right)}$ are bounded and the supports of ${\left(x^{\left(1\right)}\right)}^{T}\beta_{10}$ and ${\left(x^{\left(2\right)}\right)}^{T}\beta_{20}$ are contained in the finite interval [a,b]. Such a compactness assumption is almost always used in nonparametric regression with spline approximation. We use polynomial splines to approximate the nonparametric functions $g_{1}$ and $g_{2}$. Let $t_{0}=a<t_{1}<\cdots <t_{K^{\prime }}<b=t_{K^{\prime }+1}$ be the partitions of [a,b] into subintervals $\left[t_{k},t_{k+1}\right),k=0,\ldots ,K^{\prime }$ with $K^{\prime }$ internal knots. A polynomial spline of order d is a function whose restriction to each subinterval is a polynomial of degree d−1 and globally d−2 times continuously differentiable on [a,b]. The collection of splines with a fixed sequence of knots has a B-spline basis $\left\{B_{1}(x),\ldots ,B_{K}(x)\right\}$, with $K=K^{\prime }+d$. We assume the B-spline basis is normalized to have $\sum_{k=1}^{K}B_{k}(x)=\sqrt{K}$, although, any scaling can be used without changing the theoretical results.

Let $B_{1}(\cdot )={\left(B_{1}(\cdot ),\ldots ,B_{K_{1}}(\cdot )\right)}^{T}$ and $B_{2}(\cdot )={\left(B_{1}(\cdot ),\ldots ,B_{K_{2}}(\cdot )\right)}^{T}$, where $K_{1}=K_{1}^{\prime }+d$ and $K_{2}=K_{2}^{\prime }+d$ with number of knots $K_{1}^{\prime }$ and $K_{2}^{\prime }$ for $g_{1}$ and $g_{2}$. Then, we have $g_{k}(\cdot )\approx {B_{k}}^{T}(\cdot )\theta_{k},k=1,2$ where $\theta_{k}={\left(\theta_{k1},\ldots ,\theta_{kK_{k}}\right)}^{T},k=1,2$. As a result, we can write

(2.10) $$\mu_{ij}^{\left(1\right)}\approx {B_{1}}^{T}\left({\left(x_{ij}^{\left(1\right)}\right)}^{T}\beta_{1}\right)\theta_{1}\;\text{and}\;\mu_{ij}^{\left(2\right)}\approx {B_{2}}^{T}\left({\left(x_{ij}^{\left(2\right)}\right)}^{T}\beta_{2}\right)\theta_{2}$$

for $i=1,\ldots ,n,j=1,\ldots ,m_{i}$. By letting the number of knots increase with the sample size at an appropriate rate, the spline estimate of the unknown function can achieve the optimal nonparametric convergence rate.

## 3 Theoretical Properties

In this section, we will investigate the theoretical properties for the index parameters and the index functions. In the following we establish the large sample properties based on the marginal distribution (2.8) of the proposed BV-SIM model in (2.5). For simplicity, we assume $m_{i}\equiv m$, with the response viewed as i.i.d. data, $y_{i}∼{SALD}_{2m}\left(\mu_{i},G_{i},\gamma^{*}\right),i=1,\ldots ,n$. In (2.8), $\gamma^{*}=1_{m}⊗\gamma$ and $G_{i}={Z_{i}}^{T}\Omega Z_{i}+\Lambda$, with $\Lambda =I_{m}⊗\Sigma$. We first introduce some notations.

Let $\beta_{01}$ and $\beta_{02}$ be the true index parameters, and $g_{01}$ and $g_{02}$ the corresponding true index functions. Let $\beta_{0}={\left({\beta_{01}}^{T},{\beta_{02}}^{T}\right)}^{T},\;\beta_{0}^{\left(-1\right)}={\left({\left(\beta_{01}^{\left(-1\right)}\right)}^{T},{\left(\beta_{02}^{\left(-1\right)}\right)}^{T}\right)}^{T}$, $\mu_{i}^{0}={\left({\left(\mu_{i1}^{0}\right)}^{T},\ldots ,{\left(\mu_{im_{i}}^{0}\right)}^{T}\right)}^{T}$ with $\mu_{ij}^{0}={\left(g_{01}\left({\left(x_{ij}^{\left(1\right)}\right)}^{T}\beta_{01}\right),g_{02}\left({\left(x_{ij}^{\left(2\right)}\right)}^{T}\beta_{02}\right)\right)}^{T}$. Denote the support of $\left\{{X_{i}}^{T}\beta_{0}\right\}$ as [a,b], where $a=\min_{i}\left\{{X_{i}}^{T}\beta_{0}\right\}$ and $b={max}_{i}\left\{{X_{i}}^{T}\beta_{0}\right\}$, $X_{i}=\left(X_{i1},\ldots ,X_{im_{i}}\right)$ with $X_{ij}=\mathrm{blockdiag}\left(x_{ij}^{\left(1\right)},x_{ij}^{\left(2\right)}\right)$. Let ${ℋ}_{s}$ be the collection of all functions on the support [a,b] whose l-th order derivative satisfies the Hölder condition of the order r with s=l+r. Then, for each $g\in {ℋ}_{s}$, there exists a positive constant $C_{0}$ such that $\left|g^{\left(l\right)}(u)-g^{\left(l\right)}(v)\right|\leq C_{0}|u-v{|}^{r},\;\forall u,v\in [a,b]$. From De Boor (2001), there exists a constant C (see page 149) such that

(3.1) $$\underset{u\in [a,b]}{sup}\left|g_{k}(u)-B_{k}^{T}(u)\theta_{0k}\right|\leq CK_{k}^{-s},$$

if $g_{k}\in {ℋ}_{s}$, where $\theta_{0k}={\left(\theta_{0k1},\ldots ,\theta_{0kK_{k}}\right)}^{T},k=1,2$ are the true value of spline coefficients, which can be viewed as the best approximation coefficient vectors for $g_{k}$.

Denote $\delta ={\left(\gamma^{T},vech(\Omega {)}^{T},vech(\Sigma {)}^{T}\right)}^{T}$ and Θ as the parameter space of $\zeta ={\left(\beta^{T},\theta^{T},\delta^{T}\right)}^{T}$. Given the covariates $X_{i}$ and $Z_{i}$, let $\ell_{m}\left(\mu_{i},\delta ,y_{i}\right)$ be the log-likelihood of the marginal distribution for response $y_{i}$ in (2.8) and $\ell_{m}\left(\zeta ,y_{i}\right)≜\ell_{m}\left({W_{i}}^{T}\left({X_{i}}^{T}\beta \right)\theta ,\delta ,y_{i}\right)$ be the corresponding spline-approximated log-likelihood. Let $\delta_{0}$ be the true value of δ and $\theta_{0}={\left({\theta_{01}}^{T},{\theta_{02}}^{T}\right)}^{T}$. Define ζ^=β^T,θ^T,δ^TT as the MLE, given by

(3.2) $$\hat{\zeta }={\text{argmax}}_{\zeta }\sum_{i=1}^{n}\ell_{m}\left({W_{i}}^{\text{T}}\left({X_{i}}^{\text{T}}\beta \right)\theta ,\delta ,y_{i}\right),$$

where $W_{i}\left({X_{i}}^{T}\beta \right)=\left(W_{i1},\ldots ,W_{im_{i}}\right),\;W_{ij}=\mathrm{blockdiag}\left(B_{ij}^{\left(1\right)},B_{ij}^{\left(2\right)}\right)$ with $B_{ij}^{\left(k\right)}=B_{k}\left({\left(x_{ij}^{\left(k\right)}\right)}^{T}\beta_{k}\right),k=1,2$. Define the space of square integrable single-index functions $𝒢=\left\{g\;:\;𝔼{∥g\left({X_{i}}^{T}\beta_{0}\right)∥}^{2}<\infty \right\}$, where $g\left({X_{i}}^{T}\beta \right)={\left(g^{T}\left({X_{i1}}^{T}\beta \right),\ldots ,g^{T}\left({X_{im_{i}}}^{T}\beta \right)\right)}^{T}$ with $g\left({X_{ij}}^{T}\beta \right)={\left(g_{1}\left({\left(x_{ij}^{\left(1\right)}\right)}^{T}\beta_{1}\right),g_{2}\left({\left(x_{ij}^{\left(2\right)}\right)}^{T}\beta_{2}\right)\right)}^{T}$. Denote $C_{i}\left(\mu_{i},\delta \right)=-\partial^{2}\ell_{m}\left(\mu_{i},\delta ,y_{i}\right)/\partial \mu_{i}\partial {\mu_{i}}^{T}$ and $C_{i}^{0}=C_{i}\left(\mu_{i}^{0},\delta_{0}\right)$. Then, the projection of a 2m-dimensional random vector Γ onto 𝒢 (defined as $\left.𝔼[\Gamma ]=g\left({X_{i}}^{T}\beta_{0}\right)\right)$ is the minimizer of

$$\underset{g\in 𝒢}{min}𝔼\left[{\left(\Gamma -g\left({X_{i}}^{T}\beta_{0}\right)\right)}^{T}C_{i}^{0}\left(\Gamma -g\left({X_{i}}^{T}\beta_{0}\right)\right)\right].$$

Note, the definition of projection involves the distributions of both $X_{i},Z_{i}$ and Γ since we take the expectation over these random variables. This definition can be extended to any 2m×L matrix by column-wise projection. In the following, we list the regularity conditions (Wang et al. 2014; Lian and Liang 2013; Zhao et al. 2017) that are necessary to study the asymptotic behavior of the MLEs.

(A1) Both $g_{1}(\cdot )\in {ℋ}_{s}$ and $g_{2}(\cdot )\in {ℋ}_{s}$ for some *s* ≥ 2.
(A2) Both $x_{ij}^{\left(1\right)}$ and $x_{ij}^{\left(2\right)},i=1,\ldots ,n,j=1,\ldots ,m_{i}$, are bounded, with density supported on a convex set.
(A3) The true parameter point $\zeta_{0}$ is an interior point of the parameter space Θ.
(A4) The log-likelihood $\ell_{m}\left(\zeta ,y_{i}\right)$ is at least thrice differentiable on parameters ζ. Furthermore, the second derivatives of the likelihood function satisfy the equations
$$𝔼\left\{\left(\frac{\partial \ell_{m}\left(\zeta ,y_{i}\right)}{\partial \zeta }\right){\left(\frac{\partial \ell_{m}\left(\zeta ,y_{i}\right)}{\partial \zeta }\right)}^{T}\right\}=-𝔼\left\{\frac{\partial^{2}\ell_{m}\left(\zeta ,y_{i}\right)}{\partial \zeta \partial \zeta^{T}}\right\}.$$
  Also, there exists functions $M_{jkl}\left(y_{i}\right)$, such that
$$\left|\frac{\partial^{3}\ell_{m}\left(\zeta ,y_{i}\right)}{\partial \zeta_{j}\partial \zeta_{k}\partial \zeta_{l}}\right|\leq M_{jkl}\left(y_{i}\right)$$
  for ζ∈Θ, and $𝔼\left[M_{jkl}\left(y_{i}\right)\right]<C_{3}<+\infty$. Here $\zeta_{j}$ denotes the j-th component of ζ.
(A5) The Fisher information matrix $ℐ\left(\zeta_{0}\right)=-{\left.𝔼\left\{\frac{\partial^{2}\ell_{m}\left(\zeta ,y_{i}\right)}{\partial \zeta \partial \zeta^{T}}\right\}\right|}_{\zeta_{0}}$ satisfies the conditions
$$0<C_{1}<\lambda_{min}\left\{ℐ\left(\zeta_{0}\right)\right\}\leq \lambda_{max}\left\{ℐ\left(\zeta_{0}\right)\right\}<C_{2}<+\infty ,$$
  where $\lambda_{\text{min}}$ and $\lambda_{\text{max}}$ denote the smallest and largest eigenvalues of a matrix.
(A6) Suppose ${𝔼}_{𝒢}\left[X_{ij}diag\left\{\dot{g}\left({X_{i}}^{T}\beta_{0}\right)\right\}\right]={\left(h_{1}\left({X_{i}}^{T}\beta_{0}\right),\ldots ,h_{p_{1}+p_{2}}\left({X_{i}}^{T}\beta_{0}\right)\right)}^{T}$. Assume all $h_{j}\in {ℋ}_{s^{\prime }}$ with $s^{\prime }>1$. We also assume that
$$𝔼\left[{\left(J^{T}X_{i}diag\left\{\dot{g}\left({X_{i}}^{T}\beta_{0}\right)\right\}-{𝔼}_{𝒢}\left[J^{T}X_{i}diag\left\{\dot{g}\left({X_{i}}^{T}\beta_{0}\right)\right\}\right]\right)}^{⊗2}\right]$$
  is positive definite, where J is evaluated at $\beta_{0}$.

***Remark 1*** The smoothness condition in (A1) is a requirement to attain the best convergence rate for single-index functions approximated in the spline space. Condition (A2) is widely used in the single-index modeling literature, ensuring that the index functions are defined in a compact set and thus facilitates the technical derivations. Conditions (A3) and (A4) are two common assumptions in the literature of maximum likelihood estimation with spline approximations (Wang et al. 2011, 2014), implying that the information matrix of the likelihood function is positive definite. Condition (A5) is slightly stronger than that used in the usual asymptotic likelihood theory, however, widely used in high-dimensional likelihood estimation literature Fan and Peng (2004). Finally, Condition (A6) is related to the ‘projection’, or the ‘orthogonalization’ technique common in a semiparametric setup, which includes partially linear model (Li 2000), partially linear additive model (Lian and Liang 2013), and single-index models (Cui et al. 2011; Zhao et al. 2017).

Denote $K=max\left\{K_{1},K_{2}\right\}$, and let $r_{n}=\sqrt{K/n}+K^{-s}$. Then, we have the following result.

**Theorem 1** *Under the Conditions* (A1)–(A5), *suppose that* $K^{4}/n\to 0$, $\sqrt{n}K^{-2s+1}\to 0$, *then we have*

$$∥\hat{\beta }-\beta_{0}∥+∥\hat{\theta }-\theta_{0}∥=O_{p}\left(r_{n}\right)$$

As an immediate implication of Theorem 1, we have g^1−g1=Oprn and $∥{\hat{g}}_{2}-g_{2}∥=O_{p}\left(r_{n}\right)$.

***Remark 2*** Note that the rate of convergence for nonparametric functions is $O_{p}\left(n^{-s/(2s+1)}\right)$ if the optimal $K∼n^{1/(2s+1)}$, which is the same as that found in the nonparametric and semiparametric literature.

**Theorem 2** *Under Conditions* (A1)–(A6), *suppose that* $K^{4}/n\to 0,\sqrt{n}K^{-2s+1}\to 0$ *and* $\sqrt{n}K^{-s-s^{\prime }}\to 0$. Then, we have

$$\sqrt{n}\left({\hat{\beta }}^{\left(-1\right)}-\beta_{0}^{\left(-1\right)}\right)\overset{d}{\to }N\left(0,\Psi^{-1}\right),$$

where

$$\Psi =𝔼[\left(J^{\text{T}}X_{i}\text{diag}\left\{\dot{g}\left({X_{i}}^{\text{T}}\beta_{0}\right)\right\}-J^{\text{T}}{𝔼}_{𝒢}\left[X_{i}\text{diag}\left\{\dot{g}\left({X_{i}}^{\text{T}}\beta_{0}\right)\right\}\right]\right)\cdot C_{i}^{0}\cdot {\left(J^{\text{T}}X_{i}\text{diag}\left\{\dot{g}\left({X_{i}}^{\text{T}}\beta_{0}\right)\right\}-J^{\text{T}}{𝔼}_{𝒢}\left[X_{i}\text{diag}\left\{\dot{g}\left({X_{i}}^{\text{T}}\beta_{0}\right)\right\}\right]\right)}^{\text{T}}]$$

*and* J *is evaluated at the true* $\beta_{0}$.

Following Theorem 2 and invoking the Delta method, we have

$$\sqrt{n}\left(\hat{\beta }-\beta_{0}\right)\overset{d}{\to }N\left(0,J\Psi^{-1}J^{T}\right).$$

## 4 Maximum Likelihood Estimation

In this section, we develop the ML estimation for our BV-SIM model. We utilize EM-type algorithms for obtaining the MLE, based on two types of missing data structures in (2.6). The EM algorithm is a popular iterative algorithm for MLE in models with incomplete data (Dempster et al. 1977), where each iteration of the EM algorithm consists of two steps, the expectation (E) step and the maximization (M) step. Despite desirable features, the M-step in the EM algorithm is often difficult to implement for complicated models, and is replaced with a sequence of computationally simple conditional maximization (CM) steps, i.e. maximizing over one parameter with the other parameters held fixed. This leads to a simple extension of the EM algorithm, called the ECM algorithm (Meng and Rubin 1993).

Consider the hierarchical multivariate Laplace model in (2.6), where both $V_{i}$ and $b_{i}$ are missing data. Let $y={\left({y_{1}}^{T},\ldots ,{y_{n}}^{T}\right)}^{T},b={\left({b_{1}}^{T},\ldots ,{b_{n}}^{T}\right)}^{T},V={\left(V_{1},\ldots ,V_{n}\right)}^{T}$ and $\theta ={\left({\theta_{1}}^{T},{\theta_{2}}^{T}\right)}^{T}$. The log-likelihood for the complete data in the multivariate Laplace single-index mixed-effects model up to an additive constant can be written as

(4.1) $$\ell (\beta ,\theta ,\gamma ,\Sigma ,\Omega |y,b,V)=\ell_{1}(\beta ,\theta ,\gamma ,\Sigma |y,b,V)+\ell_{2}(\Omega |b,V),$$

where

$$\ell_{1}(\beta ,\theta ,\gamma ,\Sigma |y,b,V)=-\frac{N}{2}\text{log}|\Sigma |-\frac{1}{2}\sum_{i=1}^{n}\sum_{j=1}^{m_{i}}V_{i}^{-1}{\left(y_{ij}-{\tilde{\mu }}_{ij}-V_{i}\gamma \right)}^{\text{T}}\Sigma^{-1}\left(y_{ij}-{\tilde{\mu }}_{ij}-V_{i}\gamma \right)$$

and

$$\ell_{2}(\Omega |b,V)=-\frac{n}{2}\text{log}|\Omega |-\frac{1}{2}\text{trace}(\Omega^{-1}\sum_{i=1}^{n}V_{i}^{-1}b_{i}{b_{i}}^{\text{T}}),$$

where ${\tilde{\mu }}_{ij}$ is defined in (2.5) and $N=\sum_{i=1}^{n}m_{i}$. Note that $\ell_{1}$ can be further written as

$$\ell_{1}=-\frac{N}{2}\text{log}|\Sigma |-\frac{1}{2}\sum_{i=1}^{n}V_{i}^{-1}{\left(y_{i}-{W_{i}}^{\text{T}}\theta \right)}^{\text{T}}\Lambda_{i}^{-1}\left(y_{i}-{W_{i}}^{\text{T}}\theta \right)-\frac{1}{2}\sum_{i=1}^{n}V_{i}^{-1}{b_{i}}^{\text{T}}Z_{i}\Lambda_{i}^{-1}{Z_{i}}^{\text{T}}b_{i}\;+\sum_{i=1}^{n}V_{i}^{-1}{\left(y_{i}-{W_{i}}^{\text{T}}\theta \right)}^{\text{T}}\Lambda_{i}^{-1}{Z_{i}}^{\text{T}}b_{i}-\sum_{i=1}^{n}{\left(\gamma_{i}^{*}\right)}^{\text{T}}\Lambda_{i}^{-1}{Z_{i}}^{\text{T}}b_{i}+\sum_{i=1}^{n}{\left(y_{i}-{W_{i}}^{\text{T}}\theta \right)}^{\text{T}}\Lambda_{i}^{-1}\gamma_{i}^{*}\;-\frac{1}{2}\sum_{i=1}^{n}V_{i}{\left(\gamma_{i}^{*}\right)}^{\text{T}}\Lambda_{i}^{-1}\gamma_{i}^{*}.$$

Denote η as the full parameter vector to be estimated. We firstly compute the conditional posterior mean and variance of $b_{i}$ at the current estimate η^, leading to

$$\text{Cov}\left(b_{i}|\eta =\hat{\eta },y,V\right)=V_{i}{\left({\hat{\Omega }}^{-1}+Z_{i}{\hat{\Lambda }}_{i}^{-1}{Z_{i}}^{\text{T}}\right)}^{-1}≜V_{i}\cdot {\hat{\Delta }}_{i},\;𝔼\left(b_{i}|\eta =\hat{\eta },y,V\right)={\hat{\Delta }}_{i}Z_{i}{\hat{\Lambda }}_{i}^{-1}\left(y_{i}-{W_{i}}^{\text{T}}\hat{\theta }-V_{i}{\hat{\gamma }}_{i}^{*}\right)≜{\hat{R}}_{i1}-V_{i}{\hat{R}}_{i2},$$

for i=1,…,n, where

(4.2) Δ^i=Ω^−1+ZiΛ^i−1ZiT−1,R^1=Δ^iZiΛ^i−1yi−WiTθ^andR^2=Δ^iZiΛ^i−1γ^i*.

After obtaining the estimates of the conditional mean and conditional covariance of the random effect $b_{i}$, we proceed to calculate the expectation of $𝔼(\ell (\cdot ))={𝔼}_{V}\left\{{𝔼}_{b}[\ell (\cdot )|V]\right\}$. Define the quantities

(4.3) c^i=𝔼Vi∣η=η^,yandd^i=𝔼Vi−1∣η=η^,y,

which can be computed from (2.9), using the current estimate η^. After some simple calculations, we have

(4.4) $$Q_{1}≜𝔼\left[\ell_{1}(\cdot |y,b,V)|y,\eta =\hat{\eta }\right]\;=-\frac{N}{2}\log|\Sigma |-\frac{1}{2}\sum_{i=1}^{n}{\hat{d}}_{i}{\left(y_{i}-{W_{i}}^{\text{T}}\theta \right)}^{\text{T}}\Lambda_{i}^{-1}\left(y_{i}-{W_{i}}^{\text{T}}\theta \right)-\frac{1}{2}\sum_{i=1}^{n}{\hat{c}}_{i}{\left(\gamma_{i}^{*}\right)}^{\text{T}}\Lambda_{i}^{-1}\gamma_{i}^{*}\;-\frac{1}{2}\sum_{i=1}^{n}\mathrm{trace}\left\{Z_{i}\Lambda_{i}^{-1}{Z_{i}}^{\text{T}}\left[{\hat{d}}_{i}{\hat{R}}_{i1}{{\hat{R}}_{i1}}^{\text{T}}-{\hat{R}}_{i1}{{\hat{R}}_{i2}}^{\text{T}}-{\hat{R}}_{i2}{{\hat{R}}_{i1}}^{\text{T}}+{\hat{c}}_{i}{\hat{R}}_{i2}{{\hat{R}}_{i2}}^{\text{T}}+{\hat{\Delta }}_{i}\right]\right\}\;+\sum_{i=1}^{n}{\hat{d}}_{i}{\left(y_{i}-{W_{i}}^{\text{T}}\theta \right)}^{\text{T}}\Lambda_{i}^{-1}{Z_{i}}^{\text{T}}{\hat{R}}_{i1}-\sum_{i=1}^{n}{\left(y_{i}-{W_{i}}^{\text{T}}\theta \right)}^{\text{T}}\Lambda_{i}^{-1}\left[{Z_{i}}^{\text{T}}{\hat{R}}_{i2}-\gamma_{i}^{*}\right]\;-\sum_{i=1}^{n}{\left(\gamma_{i}^{*}\right)}^{\text{T}}\Lambda_{i}^{-1}{Z_{i}}^{\text{T}}{\hat{R}}_{i1}+\sum_{i=1}^{n}{\hat{c}}_{i}{\left(\gamma_{i}^{*}\right)}^{\text{T}}\Lambda_{i}^{-1}{Z_{i}}^{\text{T}}{\hat{R}}_{i2},$$

and

(4.5) $$Q_{2}≜𝔼\left[\ell_{2}(\cdot |y,b,V)|y,\eta =\hat{\eta }\right]\;=-\frac{n}{2}\text{log}|\Omega |-\frac{1}{2}\sum_{i=1}^{n}\;\text{trace}\left\{\Omega^{-1}\left[{\hat{d}}_{i}{\hat{R}}_{i1}{{\hat{R}}_{i1}}^{\text{T}}-{\hat{R}}_{i1}{{\hat{R}}_{i2}}^{\text{T}}-{\hat{R}}_{i2}{{\hat{R}}_{i1}}^{\text{T}}+{\hat{c}}_{i}{\hat{R}}_{i2}{{\hat{R}}_{i2}}^{\text{T}}+{\hat{\Delta }}_{i}\right]\right\}+C,$$

Next, maximizing $Q_{1}$ over parameters θ,γ,β and Σ, and maximizing $Q_{2}$ over Ω, we can obtain their estimates, which constitutes the CM-steps 1-5 in the following ECM algorithm:

E-step Given current parameter estimates, for i=1,…,n, update $c_{i}$ and $d_{i}$ using (4.3), and update ${\hat{\Delta }}_{i},{\hat{R}}_{i1}$ and ${\hat{R}}_{i2}$ by (4.2).

CM-step 1 Fix β^,γ^ and Σ^, and update θ^ by maximizing (4.4) over θ, which gives

$$\hat{\theta }={\left(\sum_{i=1}^{n}\sum_{j=1}^{m_{i}}{\hat{d}}_{i}W_{ij}{\hat{\Sigma }}^{-1}{W_{ij}}^{\text{T}}\right)}^{-1}\sum_{i=1}^{n}\sum_{j=1}^{m_{i}}W_{ij}{\hat{\Sigma }}^{-1}\left[{\hat{d}}_{i}\left(y_{ij}-{W_{ij}}^{\text{T}}\hat{\theta }-{Z_{ij}}^{\text{T}}{\hat{R}}_{i1}\right)+{Z_{ij}}^{\text{T}}{\hat{R}}_{i2}-\hat{\gamma }\right].$$

CM-step 2 Fix β^,θ^ and Σ^, update γ^ by maximizing (4.4) over γ, i.e.,

$$\hat{\gamma }=\frac{\sum_{i=1}^{n}\sum_{j=1}^{m_{i}}\left(y_{ij}-{W_{ij}}^{\text{T}}\hat{\theta }-{Z_{ij}}^{\text{T}}{\hat{R}}_{i1}+{\hat{c}}_{i}{Z_{ij}}^{\text{T}}{\hat{R}}_{i2}\right)}{\sum_{i=1}^{n}m_{i}{\hat{c}}_{i}}.$$

CM-step 3 Fix θ^,γ^ and Σ^, and update β^ by maximizing (4.4) over β. Since there is no explicit expression for the estimate of the index parameter β, we use the Newton–Raphson method to obtain $\hat{\beta }$, leading to the following iterative formula

$${\left({\hat{\beta }}^{\left(-1\right)}\right)}^{\text{new}}={\left({\hat{\beta }}^{\left(-1\right)}\right)}^{\text{old}}+{\left(\sum_{i=1}^{n}\sum_{j=1}^{m_{i}}{\hat{d}}_{i}H_{ij}{\hat{\Sigma }}^{-1}{H_{ij}}^{\text{T}}\right)}^{-1}\times \;\times \sum_{i=1}^{n}\sum_{j=1}^{m_{i}}H_{ij}{\hat{\Sigma }}^{-1}\left[{\hat{d}}_{i}\left(y_{ij}-{W_{ij}}^{\text{T}}\hat{\theta }-{Z_{ij}}^{\text{T}}{\hat{R}}_{i1}\right)+{Z_{ij}}^{\text{T}}{\hat{R}}_{i2}-\hat{\gamma }\right]$$

where Hij=J1Txij(1)B˙1Txij(1)Tβ^1oldθ^10p1−1×10p2−1×1J2Txij(2)B˙2Txij(2)Tβ^2oldθ^2, and $\dot{B}(\cdot )$ denotes the first derivative of the spline basis B(⋅).

CM-step 4 Fix $\hat{\beta },\hat{\theta }$ and γ^, and update $\hat{\Sigma }$ by maximizing (4.4) over Σ. Denote

$$\hat{D}=\sum_{i=1}^{n}\sum_{j=1}^{m_{i}}\left\{\left[{\hat{d}}_{i}\left(y_{ij}-{W_{ij}}^{\text{T}}\hat{\theta }-2{Z_{ij}}^{\text{T}}{\hat{R}}_{i1}\right)+2\left({Z_{ij}}^{\text{T}}{\hat{R}}_{i2}-\hat{\gamma }\right)\right]{\left(y_{ij}-{W_{ij}}^{\text{T}}\hat{\theta }\right)}^{\text{T}}+{\hat{c}}_{i}\hat{\gamma }{\hat{\gamma }}^{\text{T}}\right\}+\;\sum_{i=1}^{n}\sum_{j=1}^{m_{i}}{Z_{ij}}^{\text{T}}\left[{\hat{d}}_{i}{\hat{R}}_{i1}{{\hat{R}}_{i1}}^{\text{T}}-{\hat{R}}_{i1}{{\hat{R}}_{i2}}^{\text{T}}-{\hat{R}}_{i2}{{\hat{R}}_{i1}}^{\text{T}}+{\hat{c}}_{i}{\hat{R}}_{i2}{{\hat{R}}_{i2}}^{\text{T}}+{\hat{\Delta }}_{i}\right]Z_{ij}\;+\sum_{i=1}^{n}\sum_{j=1}^{m_{i}}\left({Z_{ij}}^{\text{T}}{\hat{R}}_{i1}-{\hat{c}}_{i}{Z_{ij}}^{\text{T}}{\hat{R}}_{i2}\right){\hat{\gamma }}^{\text{T}}.$$

Applying the result in Lemma 1, we obtain $\hat{\Sigma }=\frac{1}{N}\hat{D}$.

CM-step 5 Update $\hat{\Omega }$ by maximizing (4.5) over Ω, which gives

$$\hat{\Omega }=\frac{1}{n}\sum_{i=1}^{n}\left[{\hat{d}}_{i}{\hat{R}}_{i1}{{\hat{R}}_{i1}}^{\text{T}}-{\hat{R}}_{i1}{{\hat{R}}_{i2}}^{\text{T}}-{\hat{R}}_{i2}{{\hat{R}}_{i1}}^{\text{T}}+{\hat{c}}_{i}{\hat{R}}_{i2}{{\hat{R}}_{i2}}^{\text{T}}+{\hat{\Delta }}_{i}\right].$$

Repeat the above E-step and CM-steps, until all parameters achieve the desired convergence criterion. Since our estimation procedure requires initial values, we set γ^(0)=(0,0)T,Σ^(0)=I2, and the estimates of ${\hat{\beta }}_{1}^{\left(0\right)},{\hat{\beta }}_{2}^{\left(0\right)}$ and ${\hat{\Omega }}^{\left(0\right)}$ are obtained from fitting a linear mixed model via the R package lmer, where $X_{ij}=\mathrm{blockdiag}\left(x_{ij}^{\left(1\right)},x_{ij}^{\left(2\right)}\right)$ and $Z_{ij}$ are the design matrices corresponding to the fixed effects and random effects, respectively. Simulation studies (in Sect. 5) show that the above strategy works well.

## 5 Simulation Studies

In this section, we conduct extensive simulation studies using synthetic data to study the finite-sample performance of the model parameters in our proposed method (Simulation 1), and the robustness of our method when compared to existing alternatives, under data generated under various settings (Simulation 2).

### 5.1 Knots Selection

It is well-known that the performance of any spline estimation depends on the knots selection. Here, we employed Schwartz information criteria (SIC) for adaptive know selection (Ma and Song 2015; Lu 2017; Zhao et al. 2017). In view of the order $n^{1/(2s+1)}$ (of knots) to attain optimal convergence rate of nonparametric functions in 1, a sequence of knots are selected in a neighborhood of $n^{1/(2s+1)}$, such as $\left[0.5N_{s},min\left(5N_{s},n^{1/2}\right)\right]$, where $N_{s}=\left\lfloor n^{1/(2s+1)}\right\rfloor$, and s is the smoothing parameter. We choose s=2 in both simulation studies and real data application. For simplicity, we use cubic polynomial splines and the number of interior knots $K_{1}=K_{2}\equiv K$ are the same for the two nonparametric link functions. The number $K_{\text{opt}}$ corresponding to the minimum SIC value is defined as the optimal number of knots SIC(K)=−∑i=1nlogL^iK+logn×2K, where logL^iK denotes the estimated value of the log-likelihood function obtained from(2.8), with the given K knots.

### 5.2 Simulation 1: Assessing Finite-Sample Properties

Here, data is generated from the model (2.5), where the two nonparametric functions are $g_{1}(u)=2\;sin(\pi u)$ and $g_{2}(u)=8u(1-u)$, with the true index parameters $\beta_{1}=(1/\sqrt{3},-1/\sqrt{3},1/\sqrt{3}{)}^{T}$ and $\beta_{2}=(2/\sqrt{6},1/\sqrt{6},1/\sqrt{6}{)}^{T}$, respectively. Both covariates $x_{ij}^{\left(1\right)}$ and $x_{ij}^{\left(2\right)}$ are generated independently from the trivariate uniform distribution $U^{3}(0, 1)$. The random effects $b_{i}={\left({b_{i1}}^{T},{b_{i2}}^{T}\right)}^{T}$ are generated from ${SAL}_{4}(0,\Omega ,0)$, with covariance matrix

$$\Omega =\left(\begin{array}{cccc} 9 & 4.8 & 3.6 & 0.6\\ 4.8 & 4 & 2 & 1.2\\ 3.6 & 2 & 4 & 1\\ 0.6 & 1.2 & 1 & 1 \end{array}\right)$$

and the corresponding covariates $z_{ij}^{\left(1\right)}={\left(1,z_{ij1}^{\left(1\right)}\right)}^{T}$ and $z_{ij}^{\left(2\right)}={\left(1,z_{ij1}^{\left(2\right)}\right)}^{T}$, where $z_{ij1}^{\left(1\right)}$ and $z_{ij1}^{\left(2\right)}$ are generated from the standard normal distribution. The random error $\epsilon_{ij}$ is generated from ${SAL}_{2}(0,\Sigma ,\gamma )$ with $\Sigma =\left(\begin{array}{cc} 1 & 0.6\\ 0.6 & 1 \end{array}\right)$ and $\gamma =(2, 1.5{)}^{T}$. The sample size n is set to be 50, 100 and 200, and the number of cluster members $m_{i}$ in each subject is generated from the discrete uniform distribution on 5, 6,…,10. Table 1 presents the averages of bias, absolute bias, and the empirical standard error estimates for the index parameters and the skewness parameter, over 400 replications.

From Table 1, all biases are close to zero for all sample sizes, implying our proposed estimators are consistent. Moreover, the absolute biases and the standard errors are smaller with increasing sample sizes, with the estimation performance of index parameters significantly better than the skewness parameters. To further assess the estimation results, we calculate the integrated mean squared error (IMSE), defined as

$$\text{IMSE}\left(g_{l}\right)=\frac{1}{400}\sum_{s=1}^{400}\sqrt{\frac{1}{N}\sum_{i=1}^{n}\sum_{j=1}^{m_{i}}{\left\{{\hat{g}}_{l}^{\left(s\right)}\left({\left(x_{ij}^{\left(1\right)}\right)}^{\text{T}}{\hat{\beta }}_{l}\right)-g_{l}\left({\left(x_{ij}^{\left(1\right)}\right)}^{\text{T}}\beta_{l}\right)\right\}}^{2}},\;l=1,2,$$

where g^l(s)(⋅) is the spline approximation to $g_{l}(\cdot )$ in the *s*th simulation run. We report the average of the IMSE as $AIMSE=\frac{1}{2}\sum_{l=1}^{2}\;IMSE\left(g_{l}\right)$ in Table 2. For evaluating the estimation performances of the scatter matrix Σ (corresponding to the bivariate responses) and the covariance matrix Ω (for the random effects), we use the Frobenius-norm of the matrix of differences between the estimated and true values, i.e. $∥A{∥}_{F}=\sqrt{trace\left(A^{T}A\right)}$, where A is either $\hat{\Sigma }-\Sigma$ or $\hat{\Omega }-\Omega$. Simulation results, together with the root of mean square error (RMSE) for $\beta_{1},\beta_{2}$ and γ are listed in Table 2, where the RMSE for an arbitrary parameter δ is defined as RMSEδ=(δ^−δ)T(δ^−δ). It is clear from Table 2 that the finite-sample performances of our proposed estimation procedures are satisfactory, with increasing sample sizes. In sum, the simulation results show that both index parameters, the non-parametric functions, and other parameters associated with the mixed effect models are reliably estimated, thereby confirming that our proposed algorithm works well in synthetic data settings.

### 5.3 Simulation 2: Assessing Robustness, in Light of Competing Methods

Here, the data is generated similar to Simulation 1 (from a BV-SIM), except that the random effects and errors are independently generated under the following four distributional assumptions:

Case 1: $b_{i}∼N(0,\Omega ),\epsilon_{ij}∼N(0,\Sigma )$;

Case 2: $b_{i}∼t(0,\Omega ,v),\;\epsilon_{ij}∼t(0,\Sigma ,v)$;

Case 3: $b_{i}∼{SAL}_{4}(0,\Omega ,0),\epsilon_{ij}∼{SAL}_{2}(0,\Sigma ,0)$;

Case 4: $b_{i}∼0.8N(0,\Omega )+0.2N(0,10\Omega ),\;\epsilon_{ij}∼0.8N(0,\Sigma )+0.2N(0,10\Sigma )$,

for $i=1,\cdots ,n,\;j=1,\cdots ,m_{i}$,

Here, Case 1 corresponds to random effects and errors independently generated from the multivariate normal distribution. For Case 2, both are generated from the multivariate t-distribution with degree of freedom v (setting v=5). For Case 3, the random effects and errors are generated from the multivariate symmetric Laplace distribution with covariance matrix Ω and Σ, respectively. Finally, Case 4 corresponds to generating both the random terms (effects and errors) from multivariate normal mixtures. Note, for the above four cases, the bivariate clustered response is symmetric, since both the random effects and errors are generated from symmetric distributions. This is to make our approach comparable to the following two existing alternatives, (a) The bivariate normal mixed effect single-index model of Wu and Tu (2016), and (b) The bivariate mixed effect single-index model using the multivariate t-distribution, which extends the univariate linear mixed model proposal of (Pinheiro et al. 2001). In (a), penalized splines were used to approximate the nonparametric index function, whereas we use polynomial splines. At each replication, we use the same dataset to obtain the estimates from these three competing methods. We focus on the estimation of the index parameters and the index functions for the fixed effect part, with the same interpretation for all cases.

The results are summarized in Table 3. For all cases, RMSEs and AIMSEs decrease quickly as the sample size increases for all three methods. That said, our proposed method performs well for all four cases, and is significantly better than both the alternatives for Cases 3 and 4. The advantages of our method appears more prominent if we further reduce the mixing proportion of the mixture distribution in Case 4 from 0.8 to 0.7, 0.6 or 0.5 (results not reported here). In Cases 1 and 2, the performances of our method is comparable to the two others. In particular, our method performs almost similar to Pinheiro’s t-distribution method in Case 2 when n=200, while they are both better than the normal mixed-effects method of Wu and Tu (2016). To summarize, the performance of our proposed method appears to be satisfactory in all cases, and is robust to misspecified (non-Gaussian) random effects and errors, under a bivariate mixed model framework.

## 6 Application: GAAD Dataset

In this section, we illustrate our method via application to the GAAD dataset. Here, the tooth-level mean PPD and CAL measures are non-Gaussian bivariate responses representing PD status, and our objective is to evaluate the distribution of PD status for this population, and quantify the effects of various subject-level covariates such as Age (in years), body mass index (BMI), Gender (1 = Female, 0 = Male), Smoking status (1 = Smoker, 0 = Never Smoker) and glycemic level or HbA1c (1 = High/Uncontrolled), 0 = Controlled) on the PD status. For our analysis, we have n=288 subjects with complete covariate information. About 30% of the subjects are smokers. The mean age of the subjects is about 54 years with a range from 26–87 years. There is a predominance of female subjects (around 76%) in the data. Around 60% of subjects are obese (BMI ≥ 30), and 59% are with uncontrolled HbA1c. Each subject has varying number of teeth, ranging from 3 to 28, with a total of 5461 observations. A full dentition will constitute 28 teeth, however, missing tooth is very common in any oral health studies, with the actual cause of missingness mostly unknown. Hence, in order to avoid unverifiable missing data assumptions, we did not resort to missing data analysis, and present only complete case analysis.

**Fig. 2:**
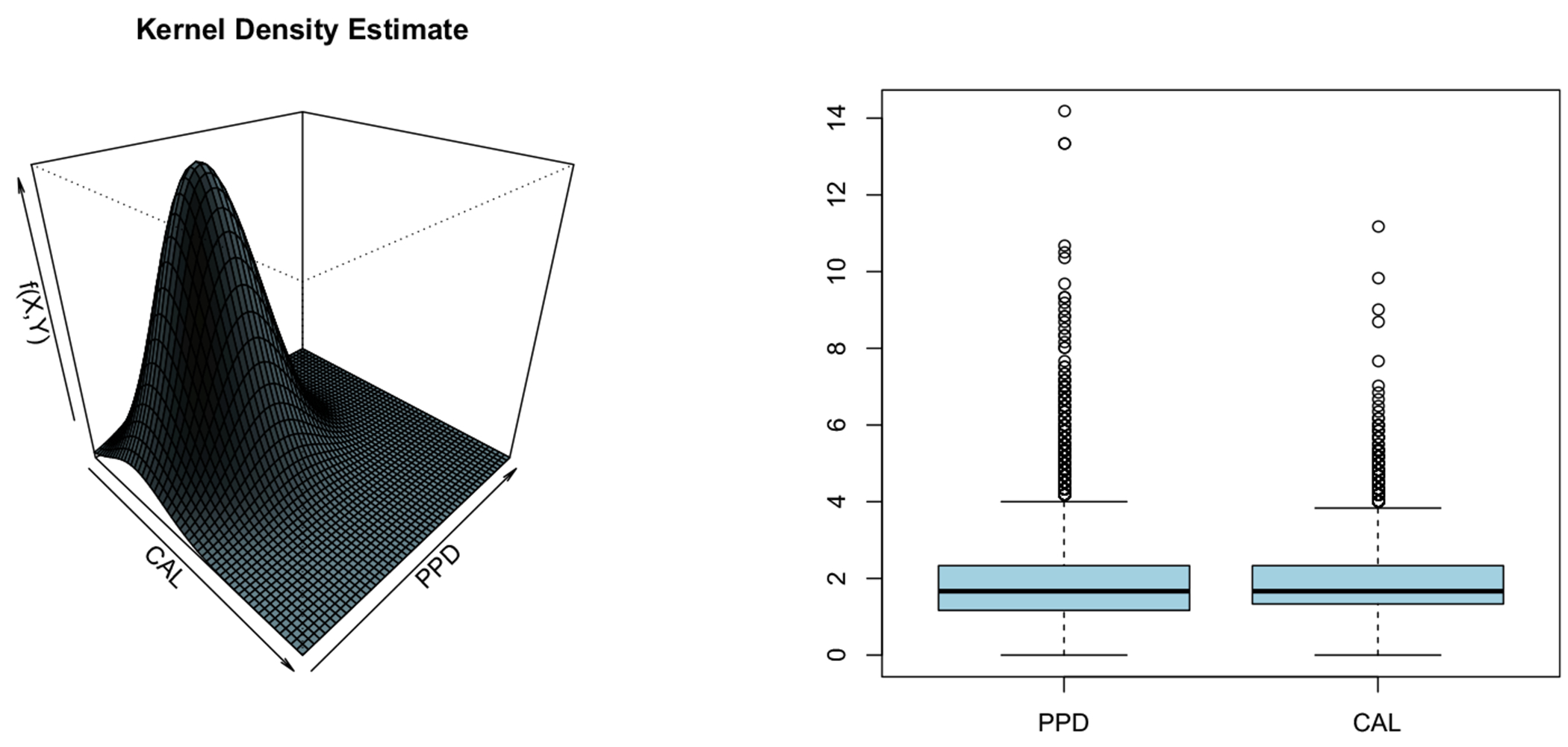
Bivariate kernel density estimate (left panel) and boxplots (right panel) for PPD and CAL responses, from the GAAD data

As part of explanatory analysis, we present the bivariate kernel density estimate of the PPD and CAL responses in Fig. 2 (left panel). The plot reveals significant (right) skewness for both responses. Also, the right panel in Fig. 2 indicates presence of possible outliers. Recent research (Zhao et al. 2018) confirmed possible non-linear relationship between oral health responses, and continuous covariates, like Age. Motivated by this, we set forward to estimate a clinically meaningful single-index structure determining PD for the subjects in this database.

**Fig. 3:**
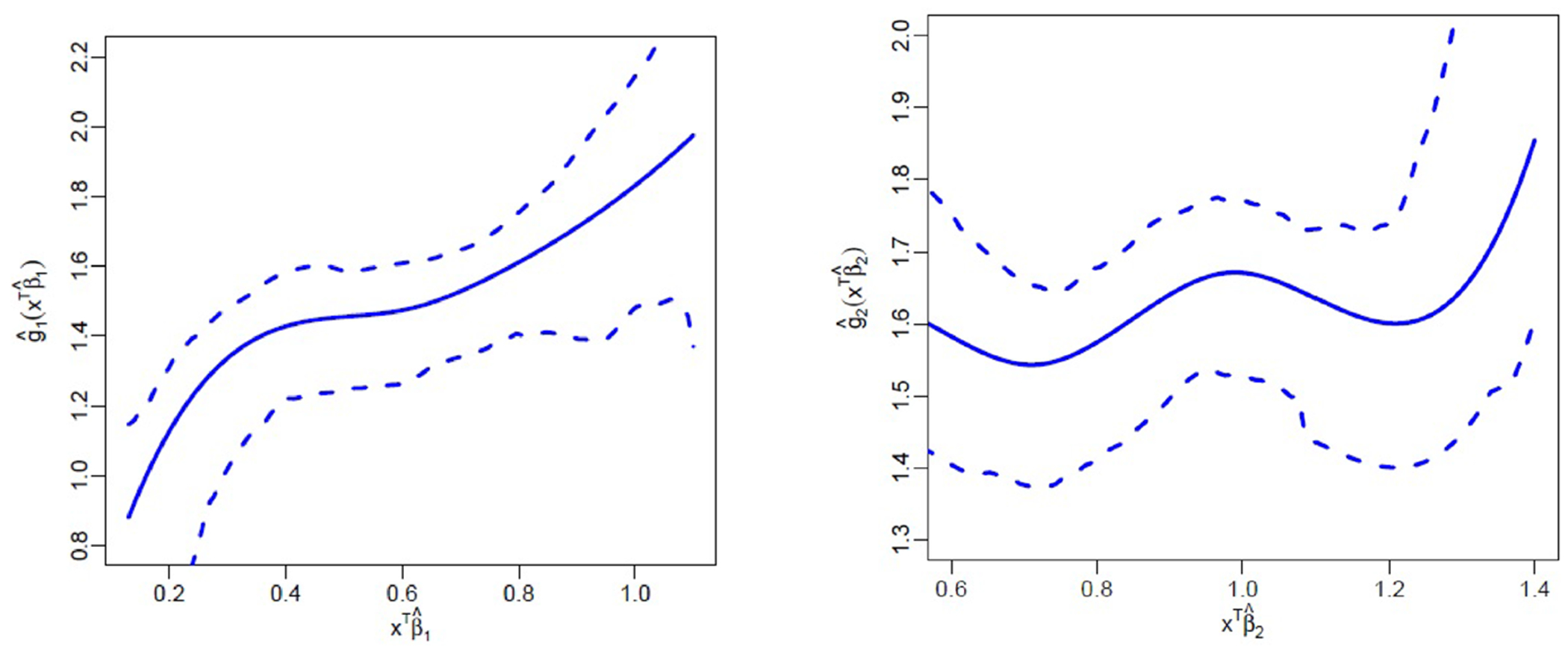
Estimated curves for the two index functions g^1 and g^2, along with the 95% confidence bands. The left and right panels correspond to PPD and CAL regressions, respectively

We consider fitting the following model to the GAAD data

$$\left\{\begin{array}{l} {PPD}_{ij}=g_{1}\left({x_{ij}}^{T}\beta_{1}\right)+{z_{ij}}^{T}b_{i1}+\epsilon_{ij1},\\ {CAL}_{ij}=g_{2}\left({x_{ij}}^{T}\beta_{2}\right)+{z_{ij}}^{T}b_{i2}+\epsilon_{ij2,} \end{array}i=1,\ldots ,288,j=1,\ldots ,m_{i}\right.,$$

where $x_{ij}={\left(x_{ij1},\ldots ,x_{ij5}\right)}^{T}$ with $x_{ij1}=\text{Age}$, $x_{ij2}=BMI,x_{ij3}=\text{Gender}$, $x_{ij4}=\text{Smoker}$, $x_{ij5}=HbA1c$ and $z_{ij}={\left(1,z_{ij1},z_{ij2},z_{ij3}\right)}^{T}$ with $z_{ij1}=\text{Gender}$, $z_{ij2}=\text{Smoker}$, $z_{ij3}=\text{HbA1c}$. We further assume $b_{i}={\left({b_{i1}}^{T},{b_{i2}}^{T}\right)}^{T}∼{SAL}_{8}(0,\Omega ,0)$ and $\epsilon_{ij}={\left(\epsilon_{ij1},\epsilon_{ij2}\right)}^{T}∼{SAL}_{2}(0,\Sigma ,\gamma )$. The estimates for index parameters, skewness parameter and their 95% confidence intervals are presented in Table 4, where the 95% confidence intervals are obtained by bootstrap resampling with 200 replications. We observe that all parameters (except $\beta_{13}$ corresponding to Gender for the PPD regression) were positive and significant. Interestingly, the estimate of Gender $\left(\beta_{13}\right)$ is negative yet significant for PPD, while, the corresponding estimate $\left(\beta_{23}\right)$ for CAL is positive and significant, implying that Gender is contributing to the index development for the two responses in opposite directions. Figure 3 presents the estimated curves corresponding to the two index functions, along with their 95% confidence bands using bootstrap method. Compared to the CAL, the 95% band is tighter for the PPD.

**Fig. 4:**
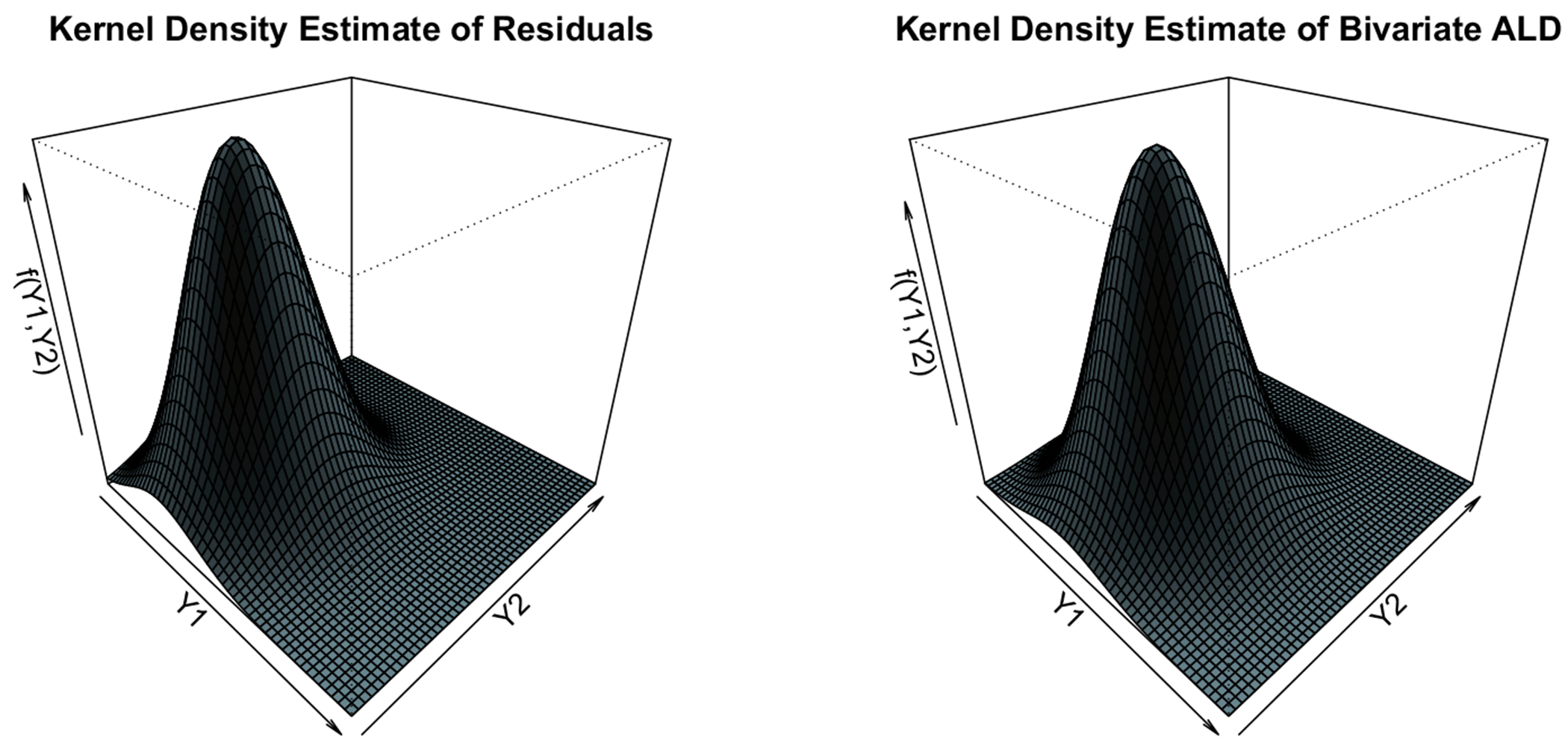
Plots of bivariate kernel density estimates from model residuals (left panel), and from random draws of n=5461 observations following $ALD(\hat{\Sigma },\hat{\gamma })$

It is immediate that the correlation between PPD and CAL are significant, implying the need to account for the crosswise correlation between the two responses, and the cluster-wise correlation of the responses within the same subject, while modeling the bivariate clustered responses. Furthermore, Fig. 4 presents the bivariate kernel density surface of the estimated residuals (left panel), and the same from random draws of n=5461 observations from the bivariate ALD density $ALD(\hat{\Sigma },\hat{\gamma })$, where $\hat{\Sigma }$ and γ^ are plugged-in estimates derived from our fit. We observe that the estimated surfaces are very similar, confirming the adequacy of model fit to the GAAD dataset.

Correlation matrices Σ and Ω are estimates as:

$$\hat{\Sigma }=\left(\begin{array}{ll} 1.2429 & 0.7937\\ 0.7937 & 0.9024 \end{array}\right)$$

and

$$\hat{\Omega }=\left(\begin{array}{cccccccc} 1.6589 & -0.0089 & -0.0461 & -0.2792 & 1.5780 & -0.1832 & -0.1815 & -0.5760\\ -0.0089 & 0.8797 & -0.4081 & 0.1553 & -0.1685 & 0.5379 & -0.0466 & 0.4289\\ -0.0461 & -0.4081 & 0.8423 & 0.3273 & -0.0808 & 0.1296 & 0.1931 & 0.1264\\ -0.2792 & 0.1553 & 0.3273 & 0.7782 & -0.4164 & 0.3802 & 0.1290 & 0.6585\\ 1.5780 & -0.1685 & -0.0808 & -0.4164 & 2.1987 & -0.8975 & -0.4840 & -0.8462\\ -0.1832 & 0.5379 & 0.1296 & 0.3802 & -0.8975 & 1.0517 & 0.3364 & 0.6420\\ -0.1815 & -0.0466 & 0.1931 & 0.1290 & -0.4840 & 0.3364 & 0.2016 & 0.1681\\ -0.5760 & 0.4289 & 0.1264 & 0.6585 & -0.8462 & 0.6420 & 0.1681 & 0.8158 \end{array}\right).$$

To further evaluate the usefulness of our proposed new model, we consider the fitted and prediction errors in light of two alternatives, denoted as “AM1” (bivariate normal, mixed effects SIM) and “AM2” (bivariate, asymmetric Laplace SIM, without random effects). We randomly partition the data into training and testing sets, where the training data is used to fit the 3 models, and the test data to evaluate the prediction errors. Using varying sizes of training and testing data, the average absolute fitted errors (AAFE), and the average absolute prediction errors (AAPE) for the two responses, based on 200 random partitions, are reported in Table 5, where

$${\text{AAFE}}_{k}=\frac{1}{\sum_{i=1}^{nb}m_{i}}\sum_{i=1}^{nb}\sum_{j=1}^{m_{i}}\left|y_{ijk}-{\hat{y}}_{ijk}\right|$$

and

$${\text{AAPE}}_{k}=\frac{1}{\sum_{i=1}^{n-nb}m_{i}}\sum_{i=1}^{n-nb}\sum_{j=1}^{m_{i}}\left|y_{ijk}-{\tilde{y}}_{ijk}\right|,$$

for k=1and2, with y^ijk, the fitted value based on training data, and ${\tilde{y}}_{ijk}$, the predicted value based on the test data, and nb denote the number of subjects in the training data.

From Table 5, we observe that our model performs the best in terms of AAFE and AAPE, for various sizes of the training and testing set. More specifically, our proposed mixed-effects SIM model is superior to the bivariate asymmetric Laplace SIM (excluding random effects), implying the necessity to account for the within-subject correlation. Furthermore, our proposed model is also better than the SIM with the usual multivariate normal specification for the random effects, thereby providing evidence of the gain in accounting for data asymmetry during modeling.

## 7 Conclusions

Derivation of useful medical indices that correlate with multiple health outcomes is an issue of significant practical importance. In this paper, we propose a single-index mixed-effects regression model for bivariate responses, where both the error term and random effect are assumed to follow multivariate asymmetric Laplace distribution. By the polynomial spline smoothing for index functions, we proposed a scalable ML estimation method based on EM-type algorithm, and study the asymptotic properties of the ML estimates under some mild conditions. Simulations and real data analysis reveal the potential of the proposed model under data asymmetry, compared to existing alternatives.

There exists a number of future directions to pursue. To further improve model fit and prediction, we can consider the joint modeling of the location, skewness, and scatter matrix, within a multivariate ALD setup. When the number of covaiates is large in both fixed effects and random effects, it is of interest to select important variables in both parts to obtain a concise model. Some existing variable selection work of linear mixed effects model are available for univariate response case; see, for example, Kinney and Dunson (2010); Bondell et al. (2010); Fan and Li (2012); Schelldorfer and Geer (2011); Pan and Huang (2014), and others. However, for the case of single-index mixed effects models for multivariate responses, there is limited work, and pursuing the variable selection is a non-trivial journey. Another extension is to consider mixed effects quantile regression (Waldmann and Kneib 2015) for bivariate responses. These will be pursued elsewhere.

## Figures and Tables

**Table 1:** Table entries are the average bias (BIAS), average absolute bias (ABIAS), and empirical standard error (ESE) estimates for *n* = 50, 100, 200, calculated over 400 replications, corresponding to Simulation 1

Parameters		$\beta_{11}$	$\beta_{12}$	$\beta_{13}$	$\beta_{21}$	$\beta_{22}$	$\beta_{23}$	$\gamma_{1}$	$\gamma_{2}$
*n* = 50	BIAS	0.0011	0.0007	−0.0011	0.0007	−0.0013	−0.0005	−0.0294	−0.0429
	ABIAS	0.0130	0.0125	0.0134	0.0061	0.0100	0.0096	0.2862	0.2479
	ESE	0.0174	0.0163	0.0169	0.0080	0.0128	0.0123	0.3596	0.3093
*n* = 100	BIAS	−0.0014	−0.0005	0.0005	−0.0001	0.0000	0.0000	−0.0417	−0.0144
	ABIAS	0.0093	0.0093	0.0085	0.0043	0.0066	0.0068	0.2299	0.1931
	ESE	0.0119	0.0120	0.0109	0.0053	0.0084	0.0085	0.2838	0.2424
*n* = 200	BIAS	0.0004	−0.0002	−0.0007	−0.0005	0.0004	0.0006	−0.0211	−0.0158
	ABIAS	0.0056	0.0055	0.0052	0.0029	0.0040	0.0041	0.1592	0.1309
	ESE	0.0072	0.0070	0.0067	0.0038	0.0052	0.0052	0.2084	0.1675

**Table 2:** Table entries are the averages of the IMSE (AIMSE), the Frobenius-norms for Σ and Ω, and the root of mean squared errors (RMSE) of the model parameters, under various sample sizes (*n* = 50, 100, 200), calculated over 400 replications, corresponding to Simulation 1

	AIMSE	$\|\hat{\Sigma }-\Sigma {\|}_{F}$	‖Ω^−Ω‖F	${\mathrm{RMSE}}_{\beta_{1}}$	${\mathrm{RMSE}}_{\beta_{2}}$	${\mathrm{RMSE}}_{\gamma }$
n=50	0.1397	0.2157	3.9259	0.0250	0.0169	0.4002
n=100	0.0976	0.1894	2.4522	0.0173	0.0115	0.3172
n=200	0.0616	0.1276	1.9059	0.0105	0.0071	0.2194

**Table 3:** Table entries are the root of mean squared errors (RMSE) of $\beta_{1}$ and $\beta_{2}$, and the Average Integrated Mean Squared Error (AIMSE) from our model and the 2 competing models (Wu and Pinheiro), for *n* = 50, 100, 200, with data generated from the 4 cases described in Sect. 5.2

		Our proposal			Wu			Pinheiro		
		${\mathrm{RMSE}}_{\beta_{1}}$	${\mathrm{RMSE}}_{\beta_{2}}$	AIMSE	${\mathrm{RMSE}}_{\beta_{1}}$	${\mathrm{RMSE}}_{\beta_{2}}$	AIMSE	${\mathrm{RMSE}}_{\beta_{1}}$	${\mathrm{RMSE}}_{\beta_{2}}$	AIMSE
*n* = 50	Case 1	0.0554	0.0358	0.0847	0.0504	0.0325	0.0780	0.0551	0.0335	0.0798
	Case 2	0.0598	0.0408	0.1333	0.0618	0.0468	0.1434	0.0593	0.0402	0.1135
	Case 3	0.0229	0.0173	0.0237	0.0439	0.0329	0.0763	0.0376	0.0265	0.0511
	Case 4	0.0673	0.0438	0.1648	0.0861	0.0571	0.2145	0.0686	0.0444	0.1666
*n* = 100	Case 1	0.0352	0.0232	0.0451	0.0308	0.0219	0.0422	0.0345	0.0225	0.0436
	Case 2	0.0382	0.0267	0.0559	0.0424	0.0313	0.0578	0.0379	0.0266	0.0450
	Case 3	0.0129	0.0109	0.0115	0.0303	0.0243	0.0374	0.0257	0.0184	0.0250
	Case 4	0.0399	0.0277	0.0776	0.0542	0.0379	0.1113	0.0434	0.0279	0.0827
*n* = 200	Case 1	0.0238	0.0167	0.0197	0.0205	0.0154	0.0188	0.0234	0.0162	0.0194
	Case 2	0.0268	0.0187	0.0253	0.0282	0.0221	0.0315	0.0267	0.0188	0.0251
	Case 3	0.0087	0.0065	0.0075	0.0207	0.0157	0.0193	0.0187	0.0122	0.0127
	Case 4	0.0312	0.0211	0.0390	0.0406	0.0278	0.0564	0.0339	0.0213	0.0414

**Table 4:** Estimates of the index parameters, the skewness parameter and their 95% confidence intervals, corresponding to the PPD and CAL responses from the GAAD study

Parameter (PPD)	Estimate	Confidence interval	Parameter (CAL)	Estimate	Confidence interval
$\beta_{11}$	0.7987	[0.7448, 0.8273]	$\beta_{21}$	0.6129	[0.4571, 0.6983]
$\beta_{12}$	0.5312	[0.4841, 0.5923]	$\beta_{22}$	0.7411	[0.6492, 0.8388]
$\beta_{13}$	−0.1219	[−0.1448, −0.1107]	$\beta_{23}$	0.0318	[0.0109, 0.0577]
$\beta_{14}$	0.1958	[0.1806, 0.2169]	$\beta_{24}$	0.1408	[0.0736, 0.2299]
$\beta_{15}$	0.1636	[0.1432, 0.1828]	$\beta_{25}$	0.2330	[0.1805, 0.3388]
$\gamma_{1}$	0.7977	[0.7037, 0.8844]	$\gamma_{2}$	0.6589	[0.5821, 0.7427]

**Table 5:** Average absolute fitted and prediction errors for our model and 2 competing models (AM1 and AM2), for the PPD and CAL responses in the GAAD data, based on 200 random partitions

Size			PPD response				CAL Response		
Training set	Test set		Our Model	AM1	AM2		Our Model	AM1	AM2
100	188	AAFE_1_	0.8670	0.9046	0.9297	AAPE_1_	0.8884	0.9339	0.9513
		AAFE_2_	0.6982	0.7005	0.7236	AAPE_2_	0.7159	0.7164	0.7364
150	138	AAFE_1_	0.8750	0.9209	0.9390	AAPE_1_	0.8813	0.9335	0.9509
		AAFE_2_	0.7024	0.7054	0.7274	AAPE_2_	0.7091	0.7138	0.7346
200	88	AAFE_1_	0.8718	0.9237	0.9406	AAPE_1_	0.8785	0.9317	0.9502
		AAFE_2_	0.6976	0.7050	0.7259	AAPE_2_	0.7126	0.7172	0.7380
250	38	AAFE_1_	0.8718	0.9265	0.9442	AAPE_2_	0.8633	0.9185	0.9408
		AAFE_2_	0.6988	0.7095	0.7309	AAPE_2_	0.6994	0.7082	0.7299

## Appendix

### Appendix 1: Lemmas

**Lemma 1** *Assume that* A *is a* d×d *positive definite matrix, then for any positive definite matrix* Σ *with dimension* d×d, *we have*

$$f(\Sigma )=|\Sigma {|}^{-n/2}\text{exp}\left\{-\frac{1}{2}\text{trace}\left(\Sigma^{-1}A\right)\right\}\leq {\left|\frac{1}{n}A\right|}^{-n/2}\text{exp}\{-\frac{nd}{2}\},$$

*if and only if* $\Sigma =\frac{1}{n}A$.

***Proof of Lemma 1*** See proofs in Anderson (1984). □

According to the MLE defined in (12), the likelihood estimating equations for β and θ can be written as

$$\sum_{i=1}^{n}{\left(\begin{array}{c} J^{\text{T}}X_{i}\text{diag}\left\{{{\dot{W}}_{i}}^{\text{T}}\left({X_{i}}^{\text{T}}\hat{\beta }\right)\hat{\theta }\right\}\\ W_{i}\left({X_{i}}^{\text{T}}\hat{\beta }\right) \end{array}\right)\frac{\partial \ell_{m}\left(\mu_{i},\hat{\delta },y_{i}\right)}{\partial \mu_{i}}|}_{\mu_{i}={W_{i}}^{\text{T}}\left({X_{i}}^{\text{T}}\hat{\beta }\right)\hat{\theta }}=0.$$

Denote ${\left.{\dot{\ell }}_{m}\left(\beta ,\theta ,\delta ,y_{i}\right)≜\frac{\partial \ell_{m}\left(\mu_{i},\delta ,y_{i}\right)}{\partial \mu_{i}}\right|}_{\mu_{i}={W_{i}}^{\text{T}}\left({X_{i}}^{T}\beta \right)\theta }$ and ${\left.{\dot{\ell }}_{m}\left(g\left({X_{i}}^{T}\beta \right),\delta ,y_{i}\right)≜\frac{\ell_{m}\left(\mu_{i},\delta ,y_{i}\right)}{\partial \mu_{i}}\right|}_{\mu_{i}=g\left({X_{i}}^{T}\beta \right)}$. Then we have the following Lemma 2.

**Lemma 2** *Assuming Conditions* (*A*1)–(*A*6) *hold, we have*

(18)
$$∥\sum_{i=1}^{n}\begin{array}{l} {}[\left(\begin{array}{c} J^{\text{T}}X_{i}\text{diag}\left\{{{\dot{W}}_{i}}^{\text{T}}\left({X_{i}}^{\text{T}}\beta \right)\theta \right\}\\ W_{i}\left({X_{i}}^{\text{T}}\beta \right) \end{array}\right)-\left(\begin{array}{c} J^{\text{T}}X_{i}\text{diag}\left\{{{\dot{W}}_{i}}^{\text{T}}\left({X_{i}}^{\text{T}}\beta_{0}\right)\theta_{0}\right\}\\ W_{i}\left({X_{i}}^{\text{T}}\beta_{0}\right) \end{array}\right)]\|{\dot{\ell }}_{m}\left(\beta ,\theta ,\delta ,y_{i}\right) \end{array}\;=o_{p}(\sqrt{n})$$

and

(19)
$$\left\|\sum_{i=1}^{n}\begin{array}{l} \left[\left(\begin{array}{c} J^{\text{T}}X_{i}\text{diag}\left\{{{\dot{W}}_{i}}^{\text{T}}\left({X_{i}}^{\text{T}}\beta \right)\theta \right\}\\ W_{i}\left({X_{i}}^{\text{T}}\beta \right) \end{array}\right)-\left(\begin{array}{c} J^{\text{T}}X_{i}\text{diag}\left\{{{\dot{W}}_{i}}^{\text{T}}\left({X_{i}}^{\text{T}}\beta_{0}\right)\theta_{0}\right\}\\ W_{i}\left({X_{i}}^{\text{T}}\beta_{0}\right) \end{array}\right)\right]{\dot{\ell }}_{m}\left(g\left({X_{i}}^{\text{T}}\beta_{0}\right),\delta_{0},y_{i}\right) \end{array}\;=o_{p}(\sqrt{n}\right)$$

*uniformly over* $∥\beta^{\left(-1\right)}-\beta_{0}^{\left(-1\right)}∥+∥\theta -\theta_{0}∥+∥\delta -\delta_{0}∥\leq Cr_{n}$.

***Proof of Lemma 2*** We firstly prove (19). To obtain the bound, we only need to calculate the conditional variance of the left term in (19) since the conditional expection $𝔼\left({\dot{\ell }}_{m}\left(g\left({X_{i}}^{T}\beta_{0}\right),\delta_{0},y_{i}\right)|X_{i},Z_{i}\right)=0$. By the Condition (A4), the eigenvalues of the conditional variance for $𝕍ar\left({\dot{\ell }}_{m}\left(g\left({X_{i}}^{T}\beta_{0}\right),\delta_{0},y_{i}\right)|X_{i},Z_{i}\right)$ are bounded, hence we only need to obtain the bound of $∥\begin{array}{c} J^{T}X_{i}diag\left\{{{\dot{W}}_{i}}^{T}\left({X_{i}}^{T}\beta \right)\theta -{{\dot{W}}_{i}}^{T}\left({X_{i}}^{T}\beta_{0}\right)\theta_{0}\right\}\\ W_{i}\left({X_{i}}^{T}\beta \right)-W_{i}\left({X_{i}}^{T}\beta_{0}\right) \end{array}∥$.

By the properties of spline basis, we have

$$\left|{W_{i}}^{\text{T}}\left({X_{i}}^{T}\beta \right)\theta -{W_{i}}^{\text{T}}\left({X_{i}}^{T}\beta_{0}\right)\theta_{0}\right|\leq \left|{{\dot{W}}_{i}}^{T}\left({X_{i}}^{T}\beta^{*}\right)\theta {X_{i}}^{T}\left(\beta -\beta_{0}\right)\right|+\left|{W_{i}}^{\text{T}}\left({X_{i}}^{T}\beta \right)\left(\theta -\theta_{0}\right)\right|\;\leq CK^{1/2}\left(∥\beta -\beta_{0}∥+∥\theta -\theta_{0}∥\right)$$

and

$$\left|{{\dot{W}}_{i}}^{T}\left({X_{i}}^{T}\beta \right)\theta -{{\dot{W}}_{i}}^{T}\left({X_{i}}^{T}\beta_{0}\right)\theta_{0}\right|\leq \left|{\ddot{W}}_{i}^{T}\left({X_{i}}^{T}\beta^{**}\right)\theta {X_{i}}^{T}\left(\beta -\beta_{0}\right)\right|+\left|{{\dot{W}}_{i}}^{T}\left({X_{i}}^{T}\beta \right)\left(\theta -\theta_{0}\right)\right|\;\leq CK^{3/2}\left(∥\beta -\beta_{0}∥+∥\theta -\theta_{0}∥\right),$$

where both $\beta^{*}$ and $\beta^{**}$ lies on the line segment connecting β and $\beta_{0}$. As a result,

(20)
$$\|\begin{array}{c} J^{\text{T}}X_{i}diag\left\{{{\dot{W}}_{i}}^{\text{T}}\left({X_{i}}^{\text{T}}\beta \right)\theta -{{\dot{W}}_{i}}^{\text{T}}\left({X_{i}}^{\text{T}}\beta_{0}\right)\theta_{0}\right\}\\ W_{i}\left({X_{i}}^{\text{T}}\beta \right)-W_{i}\left({X_{i}}^{\text{T}}\beta_{0}\right) \end{array}\|\leq CK^{3/2}r_{n}.$$

Then the order of (19) is $O_{p}\left(\sqrt{n}K^{3/2}r_{n}\right)=o_{p}(\sqrt{n})$ since d>2.

We next prove (18). By the Taylor’s expansion and regularity conditions, it is clear that

$$∥{\dot{\ell }}_{m}\left(\beta ,\theta ,\delta ,y_{i}\right)-{\dot{\ell }}_{m}\left(g\left({X_{i}}^{T}\beta_{0}\right),\delta_{0},y_{i}\right)∥=O_{p}\left(r_{n}\right).$$

Applying the results of (19) and (20), the order of (18) is $o_{p}(\sqrt{n})+O_{p}\left(nK^{3/2}r_{n}^{2}\right)=o_{p}(\sqrt{n})$.

**Lemma 3** *Assume that Condition* (*A*1)–(*A*6) *hold, the singular values of the matrix*

$$\frac{1}{n}\sum_{i=1}^{n}\left(\begin{array}{c} J^{\text{T}}X_{i}\text{diag}\left\{{{\dot{W}}_{i}}^{\text{T}}\left({X_{i}}^{\text{T}}\beta_{0}\right)\theta_{0}\right\}\\ W_{i}\left({X_{i}}^{\text{T}}\beta_{0}\right) \end{array}\right)C_{i}^{0}\left(\begin{array}{c} J^{\text{T}}X_{i}\text{diag}\left\{{{\dot{W}}_{i}}^{\text{T}}\left({X_{i}}^{\text{T}}\beta_{0}\right)\theta_{0}\right\}\\ W_{i}\left({X_{i}}^{\text{T}}\beta_{0}\right) \end{array}\right)\text{T}$$

are bounded and bounded away from zero with probability approaching one.

***Proof of Lemma 3*** Note that we can replace ${{\dot{W}}_{i}}^{T}\left({X_{i}}^{T}\beta_{0}\right)\theta_{0}$ with $\dot{g}\left({X_{i}}^{T}\beta_{0}\right)$ in above expression with only a difference of $o_{p}(1)$ since $∥{{\dot{W}}_{i}}^{T}\left({X_{i}}^{T}\beta_{0}\right)\theta_{0}-\dot{g}\left({X_{i}}^{T}\beta_{0}\right)∥\leq CK^{-s+1}$. Therefore we next only need to show that the eigenvalues of

$$\frac{1}{n}\sum_{i=1}^{n}\left(\begin{array}{c} J^{\text{T}}X_{i}\text{diag}\left\{\dot{g}\left({X_{i}}^{\text{T}}\beta_{0}\right)\right\}\\ W_{i}\left({X_{i}}^{\text{T}}\beta_{0}\right) \end{array}\right)C_{i}^{0}\left(\begin{array}{c} J^{\text{T}}X_{i}\text{diag}\left\{\dot{g}\left({X_{i}}^{\text{T}}\beta_{0}\right)\right\}\\ W_{i}\left({X_{i}}^{\text{T}}\beta_{0}\right) \end{array}\right)\text{T}$$

are bounded and bounded away from zero.

By the Condition (A6), there exists a $\left(p_{1}+p_{2}\right)\times \left(K_{1}+K_{2}\right)$ matrix $\Pi_{0}$ such that

$$∥{𝔼}_{𝒢}\left[X_{i}diag\left\{\dot{g}\left({X_{i}}^{T}\beta_{0}\right)\right\}\right]-\Pi_{0}{\dot{W}}_{i}\left({X_{i}}^{T}\beta_{0}\right)∥\leq CK^{-s^{\prime }}.$$

It is obvious that the singular values of $\left(\begin{array}{cc} I & -\Pi_{0}\\ 0 & I \end{array}\right)$ are bounded and bounded away from zero. Thus, by pre-/post-multiplying this matrix, we only need to prove that the singular values of

$$\frac{1}{n}\sum_{i=1}^{n}\left(\begin{array}{cc} J^{\text{T}}X_{i}\text{diag}\left\{\dot{g}\left({X_{i}}^{\text{T}}\beta_{0}\right)\right\}-J^{\text{T}}\Pi_{0}{\dot{W}}_{i}\left({X_{i}}^{\text{T}}\beta_{0}\right) & W_{i}\left({X_{i}}^{\text{T}}\beta_{0}\right) \end{array}\right)C_{i}^{0}\;\times (\begin{array}{cc} J^{\text{T}}X_{i}\text{diag}\left\{\dot{g}\left({X_{i}}^{\text{T}}\beta_{0}\right)\right\}-J^{\text{T}}\Pi_{0}{\dot{W}}_{i}\left({X_{i}}^{\text{T}}\beta_{0}\right) & W_{i}\left({X_{i}}^{\text{T}}\beta_{0}\right) \end{array})\text{T}$$

are bounded and bounded away from zero. Apply the approximation of splines again, we only need to show that the singular values of

$$\frac{1}{n}\sum_{i=1}^{n}\left(\begin{array}{c} J^{\text{T}}X_{i}\mathrm{diag}\left\{\dot{g}\left({X_{i}}^{\text{T}}\beta_{0}\right)\right\}-J^{\text{T}}{𝔼}_{𝒢}\left[X_{i}\mathrm{diag}\left\{\dot{g}\left({X_{i}}^{\text{T}}\beta_{0}\right)\right\}\right]\\ W_{i}\left({X_{i}}^{\text{T}}\beta_{0}\right) \end{array}\right)C_{i}^{0}\;\times (\begin{array}{c} J^{\text{T}}X_{i}\mathrm{diag}\left\{\dot{g}\left({X_{i}}^{\text{T}}\beta_{0}\right)\right\}-J^{\text{T}}{𝔼}_{𝒢}\left[X_{i}\mathrm{diag}\left\{\dot{g}\left({X_{i}}^{\text{T}}\beta_{0}\right)\right\}\right]\\ W_{i}\left({X_{i}}^{\text{T}}\beta_{0}\right) \end{array})\text{T}$$

are bounded, and bounded away from zero. By the law of large numbers, we only need to show its expectation has eigenvalues bounded and bounded away from zero. This is true by checking Conditions (A5) and (A6). □

***Proof of Theorem 1*** By Lemma 2 and Taylor’s expansion, it is easy to show

(21)
$$\sum_{i=1}^{n}\left(\begin{array}{c} J^{\text{T}}X_{i}\text{diag}\left\{{{\dot{W}}_{i}}^{\text{T}}\left({X_{i}}^{\text{T}}\beta_{0}\right)\theta_{0}\right\}\\ W_{i}\left({X_{i}}^{\text{T}}\beta_{0}\right) \end{array}\right){\dot{\ell }}_{m}\left(\beta ,\theta ,\delta ,y_{i}\right)\;=\sum_{i=1}^{n}\left(\begin{array}{c} J^{\text{T}}X_{i}\text{diag}\{{{\dot{W}}_{i}}^{\text{T}}({X_{i}}^{\text{T}}\beta_{0})\theta_{0}\}\\ W_{i}\left({X_{i}}^{\text{T}}\beta_{0}\right) \end{array}\right){\dot{\ell }}_{m}\left(g\left({X_{i}}^{\text{T}}\beta_{0}\right),\delta_{0},y_{i}\right)\;-\sum_{i=1}^{n}\left(\begin{array}{c} J^{\text{T}}X_{i}\text{diag}\left\{{{\dot{W}}_{i}}^{\text{T}}\left({X_{i}}^{\text{T}}\beta_{0}\right)\theta_{0}\right\}\\ W_{i}\left({X_{i}}^{\text{T}}\beta_{0}\right) \end{array}\right)C_{i}^{0}\left(\begin{array}{c} J^{\text{T}}X_{i}\text{diag}\left\{{{\dot{W}}_{i}}^{\text{T}}\left({X_{i}}^{\text{T}}\beta_{0}\right)\theta_{0}\right\}\\ W_{i}\left({X_{i}}^{\text{T}}\beta_{0}\right) \end{array}\right)\text{T}\left(\begin{array}{c} \beta^{\left(-1\right)}-\beta^{\left(-1\right)}\\ \theta -\theta_{0} \end{array}\right)\;+o_{p}(\sqrt{n})+O_{p}\left(nr_{n}\right)$$

if $∥\beta^{\left(-1\right)}-\beta_{0}^{\left(-1\right)}∥+∥\theta -\theta_{0}∥+∥\delta -\delta_{0}∥=O_{p}\left(r_{n}\right)$.

By direct variance calculation

(22)
$$\sum_{i=1}^{n}\left(\begin{array}{c} J^{\text{T}}X_{i}\text{diag}\left\{{{\dot{W}}_{i}}^{\text{T}}\left({X_{i}}^{\text{T}}\beta_{0}\right)\theta_{0}\right\}\\ W_{i}\left({X_{i}}^{\text{T}}\beta_{0}\right) \end{array}\right){\dot{\ell }}_{m}\left(g\left({X_{i}}^{\text{T}}\beta_{0}\right),\delta_{0},y_{i}\right)=O_{p}(\sqrt{nK}).$$

Moreover, by Lemma 3, the singular values of the matrix

$$\frac{1}{n}\sum_{i=1}^{n}\left(\begin{array}{c} J^{\text{T}}X_{i}\text{diag}\left\{{{\dot{W}}_{i}}^{\text{T}}\left({X_{i}}^{\text{T}}\beta_{0}\right)\theta_{0}\right\}\\ W_{i}\left({X_{i}}^{\text{T}}\beta_{0}\right) \end{array}\right)C_{i}^{0}\left(\begin{array}{c} J^{\text{T}}X_{i}\text{diag}\left\{{{\dot{W}}_{i}}^{\text{T}}\left({X_{i}}^{\text{T}}\beta_{0}\right)\theta_{0}\right\}\\ W_{i}\left({X_{i}}^{\text{T}}\beta_{0}\right) \end{array}\right)\text{T}$$

are bounded and bounded away from zero with probability approaching one.

Combining the above results of (21) and (22) together with Lemma 2, if choosing L sufficiently large enough for $∥\beta^{\left(-1\right)}-\beta_{0}^{\left(-1\right)}∥+∥\theta -\theta_{0}∥+∥\delta -\delta_{0}∥=Lr_{n}$, we have

$$P(\|\sum_{i=1}^{n}\left(\begin{array}{c} J^{\text{T}}X_{i}\text{diag}\left\{{{\dot{W}}_{i}}^{\text{T}}\left({X_{i}}^{\text{T}}\beta \right)\theta \right\}\\ W_{i}\left({X_{i}}^{\text{T}}\beta \right) \end{array}\right){\dot{\ell }}_{m}\left(\beta ,\theta ,\delta ,y_{i})\right\|>\|\sum_{i=1}^{n}\left(\begin{array}{c} J^{\text{T}}X_{i}\text{diag}\left\{{{\dot{W}}_{i}}^{\text{T}}\left({X_{i}}^{\text{T}}\beta_{0}\right)\theta_{0}\right\}\\ W_{i}\left({X_{i}}^{\text{T}}\beta_{0}\right) \end{array}\right){\dot{\ell }}_{m}\left(\beta_{0},\theta_{0},\delta_{0},y_{i}\right)\|)\to 1.$$

Thus we can conclude that β^−β0+θ^−θ0=Oprn. □

***Proof of Theorem 2*** Denote $T={\left(T_{1},\ldots ,T_{n}\right)}^{T},D=\mathrm{diag}\left(C_{1}^{0},\ldots ,C_{n}^{0}\right)$ and define the “projection matrix” $P=T{\left(T^{T}DT\right)}^{-1}T^{T}D$, where $T_{i}=W_{i}\left({X_{i}}^{T}\beta_{0}\right)$. Let $X_{i}^{*}=J^{T}X_{i}\mathrm{diag}\left\{{{\dot{W}}_{i}}^{T}\left({X_{i}}^{T}\beta_{0}\right)\theta_{0}\right\},X^{*}={\left(X_{1}^{*},\ldots ,X_{n}^{*}\right)}^{T}$ and ${\tilde{X}}^{*}=(I-P)X^{*}$. Then we can write ${\tilde{X}}_{i}^{*}=X_{i}^{*}-AT_{i}$ where $A=X^{*T}DT{\left(T^{T}DT\right)}^{-1}$. Let $\tilde{A}=\left(\begin{array}{ll} I & A\\ 0 & I \end{array}\right)$. By the Lemma 2 and the proof shown in Theorem 1, we have

$$\underset{\|\beta^{\left(-1\right)}-\beta_{0}^{\left(-1\right)}\|+\|\theta -\theta_{0}\|+\|\delta -\delta_{0}\|\leq {\mathrm{Cr}}_{n}}{\text{sup}}\|\tilde{A}\sum_{i=1}^{n}\left(\begin{array}{c} {\tilde{X}}_{i}^{*}\\ T_{i} \end{array}\right){\dot{\ell }}_{m}\left(\beta ,\theta ,\delta ,y_{i}\right)\;-\tilde{A}\sum_{i=1}^{n}\left(\begin{array}{c} {\tilde{X}}_{i}^{*}\\ T_{i} \end{array}\right){\dot{\ell }}_{m}\left(g\left({X_{i}}^{\text{T}}\beta_{0}\right),\delta_{0},y_{i}\right)\;+\tilde{A}\sum_{i=1}^{n}\left(\begin{array}{c} {\tilde{X}}_{i}^{*}\\ T_{i} \end{array}\right)C_{i}^{0}\left[\left({\tilde{X}}_{i}^{*\text{T}},{T_{i}}^{\text{T}}\right)\left(\begin{array}{c} \beta^{\left(-1\right)}-\beta_{0}^{\left(-1\right)}\\ \theta -\theta_{0}+A^{\text{T}}\left(\beta^{\left(-1\right)}-\beta_{0}^{\left(-1\right)}\right) \end{array}\right)+R_{i}\right]\|\;=o_{p}(\sqrt{n}),$$

where $R_{i}={W_{i}}^{\text{T}}\left({X_{i}}^{T}\beta \right)\theta -g\left({X_{i}}^{T}\beta_{0}\right)$.

By parameter transformation, we can write $\theta -\theta_{0}+A^{T}\left(\beta^{\left(-1\right)}-\beta_{0}^{\left(-1\right)}\right)$ as $\vartheta -\vartheta_{0}$. Further denote

$$U(\beta ,\vartheta )≜\tilde{A}\sum_{i=1}^{n}\left(\begin{array}{c} {\tilde{X}}_{i}^{*}\\ T_{i} \end{array}\right){\dot{\ell }}_{m}\left(g\left({X_{i}}^{\text{T}}\beta_{0}\right),\delta_{0},y_{i}\right)\;-\sum_{i=1}^{n}\left(\begin{array}{c} {\tilde{X}}_{i}^{*}\\ T_{i} \end{array}\right)C_{i}^{0}\left[\left({\tilde{X}}_{i}^{*\text{T}},{T_{i}}^{\text{T}}\right)\left(\begin{array}{c} \beta^{\left(-1\right)}-\beta_{0}^{\left(-1\right)}\\ \vartheta -\vartheta_{0} \end{array}\right)+R_{i}\right],$$

and the first $p_{1}+p_{2}-2$ and the last $K_{1}+K_{2}$ equations of U(β,ϑ) as $U_{1}(\beta ,\vartheta )$ and $U_{2}(\beta ,\vartheta )$, respectively. Let

$${\tilde{\beta }}^{\left(-1\right)}=\beta_{0}^{\left(-1\right)}+{\left(\sum_{i=1}^{n}{\tilde{X}}_{i}^{*}C_{i}^{0}{\tilde{X}}_{i}^{*\text{T}}\right)}^{-1}\sum_{i=1}^{n}{\tilde{X}}_{i}^{*}{\dot{\ell }}_{m}\left(g\left({X_{i}}^{\text{T}}\beta_{0}\right),\delta_{0},y_{i}\right).$$

It is easy to see that

$$\|\frac{1}{n}\sum_{i=1}^{n}{\tilde{X}}_{i}^{*}C_{i}^{0}{\tilde{X}}_{i}^{*\text{T}}-\Psi \|=o_{p}(1),$$

and by the central limit theorem, we have

$$\sqrt{n}\left({\tilde{\beta }}^{\left(-1\right)}-\beta_{0}^{\left(-1\right)}\right)\overset{d}{\to }N\left(0,\Psi_{1}^{-1}\right).$$

In the following, we only need to show $∥{\hat{\beta }}^{\left(-1\right)}-{\tilde{\beta }}_{0}^{\left(-1\right)}∥=o_{p}(1/\sqrt{n})$.

For any β satisfying $∥\beta^{\left(-1\right)}-\beta_{0}^{\left(-1\right)}∥=\varepsilon /\sqrt{n},\forall \varepsilon >0$, similar to the proof of Lemma A.6 in Zhao et al. (2017), we can show that

$$\|\sum_{i=1}^{n}{\tilde{X}}_{i}^{*}C_{i}^{0}R_{i}\|=o_{p}(\sqrt{n})\;\text{and}\;\|\sum_{i=1}^{n}T_{i}C_{i}^{0}R_{i}\|=o_{p}(n),$$

which lead to

U2(β,ϑ^)−U2(β˜,ϑ^)=op(n).

Furthermore, note that

$$\sum_{i=1}^{n}{\tilde{X}}_{i}^{*}C_{i}^{0}{T_{i}}^{\text{T}}=\sum_{i=1}^{n}\left(X_{i}^{*}-X^{*\text{T}}DT{\left(T^{\text{T}}DT\right)}^{-1}T_{i}\right)D_{i}{T_{i}}^{\text{T}}=0,$$

and $U_{1}(\beta ,\vartheta )$ is a linear function of β up to a $o_{p}(\sqrt{n})$ term. Consequently,

U1(β,ϑ^)≥Cn‖β−β˜‖+op(n).

As a result, we have

$$\left\|\tilde{A}U(\beta ,\hat{\vartheta })\|\geq C\varepsilon \sqrt{n}\;\text{while}\;\|\tilde{A}U(\tilde{\beta },\hat{\vartheta })\|=o_{p}(\sqrt{n}\right)$$

since the eigenvalues of $\tilde{A}{\tilde{A}}^{T}$ are bounded and bounded away from zero with probability approaching 1. Then we can conclude that β^(−1)−β˜0(−1)=op(1/n) holds. □
